# Palladium Metal Nanocomposites Based on PEI-Functionalized Nitrogen-Doped Graphene Quantum Dots: Synthesis, Characterization, Density Functional Theory Modeling, and Cell Cycle Arrest Effects on Human Ovarian Cancer Cells

**DOI:** 10.1021/acsomega.3c10324

**Published:** 2024-03-06

**Authors:** Buket Altinok Gunes, Omer Faruk Kirlangic, Murat Kilic, Asuman Sunguroglu, Taner Ozgurtas, Ecem Kaya Sezginer, Bahadir Boyacioglu, Huseyin Unver, Mustafa Yildiz

**Affiliations:** † Vocational School of Health Services, Ankara University, Ankara 06290, Turkiye; ‡ Department of Medical Biology, School of Medicine, Ankara University, Ankara 06620, Turkiye; § Department of Medical Biochemistry, Gulhane School of Medicine, University of Health Sciencies, Ankara 06018, Turkiye; ∥ Department of Biochemistry, Faculty of Pharmacy, Ankara University, Ankara 06100, Turkiye; ⊥ Department of Physics, Faculty of Science, Ankara University, Ankara 06100, Turkiye; # Department of Chemistry, Faculty of Sciences, Canakkale Onsekiz Mart University, Canakkale 17100, Turkiye

## Abstract

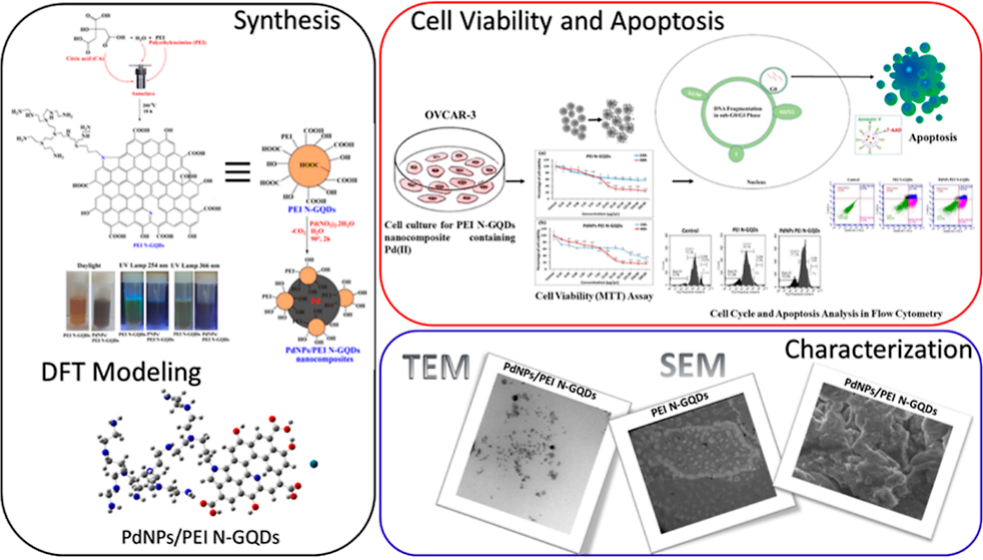

In this study, the synthesis, characterization, density functional theory calculations (DFT), and effect of polyethylenimine (PEI)-functionalized nitrogen-doped graphene quantum dots (PEI N-GQDs) and their palladium metal nanoparticles nanocomposites (PdNPs/PEI N-GQDs) on cancer cells were extensively investigated. The focus also includes investigating their cytotoxic and apoptotic effects on ovarian cancer cells, which pose a serious risk to women’s health and have high death rates from delayed diagnosis, inadequate response to treatment, and decreased survival. Graphene quantum dots and their palladium nanocomposites were differentially effective against ovarian cancer cell lines. In particular, the smaller particle size and morphology of PdNPs/PEI N-GQDs nanocomposites compared with PEI N-GQDs probably enhance their activity through highly improved uptake by cells. These findings emphasize the importance of particle size in composite drugs for efficient cancer treatment. DFT results revealed that the Pd-containing nanocomposite, with a smaller highest occupied molecular orbital–lowest unoccupied molecular orbital gap, exhibited higher reactivity and anticancer effects in human ovarian cancer cell line, OVCAR-3. Significantly, the application of nanocomposites to ovarian cancer cells initiated apoptosis, offering valuable insights into the intricate interplay between nanomaterials and cancer biology.

## 1 Introduction

Quantum dots (QDs) have attracted great interest in the field of nanotechnology, which has become a fascinating field in recent years.^1^ In particular, graphene quantum dots (GQDs), which are a subclass of QDs and more widely used in applications ranging from sensing and imaging to drug delivery and energy storage, are derived from graphene sheets and exhibit a combination of carbon dot-like and graphene-like properties, making them suitable for a wide range of applications.^2^ As novel materials with distinctive properties, GQDs can have their attributes deliberately modified through heteroatom doping, thereby facilitating their use in a broad spectrum of innovative applications. N-GQDs, unlike GQDs without nitrogen, produce blue light and exhibit electrocatalytic performance similar to that of a Pt/C catalyst in the oxygen reduction reaction (ORR) under alkaline conditions.^3^ Beyond serving as catalysts for the ORR in fuel cells without the need for metals, the exceptional luminescent properties of N-GQDs make them suitable for applications in biomedical imaging and various optoelectronic applications.

N-GQDs show great promise in the fields of catalysis and photoluminescence. The selection of nitrogen dopants in N-GQDs allows for the modulation of the overall nitrogen content and the arrangement of functional groups, providing control over the emission wavelengths and lifetimes of the photoluminescence. Elevated concentrations of amine groups typically result in a redshift in emission and shorter lifetimes, whereas pyridinic groups cause a blueshift with longer lifetimes. In comparison to electrocatalysts employed in the reduction of oxygen to hydrogen peroxide, a significant chemical widely utilized in industrial applications, N-GQDs demonstrate both a low overpotential and high selectivity for the two-electron oxygen reduction process.^4^ These nanocomposites represent the convergence of cutting-edge materials science and biotechnology and offer a unique combination of properties that make them ideal candidates for targeted cancer therapy.^5,6^ Cancer, nowadays a major global public health problem,^7^ continues to pose a formidable challenge in modern medicine, requiring innovative and diverse approaches for its diagnosis and treatment. In recent years, nanotechnology has emerged as a promising frontier in the search for more effective cancer therapies. Among numerous nanomaterials being investigated for biological applications, and especially cancer therapy, N-GQDs stand out due to their excellent biocompatibility and photoluminescence properties, as well as serving as valuable tools due to their unique properties that enhance drug delivery and imaging in the context of cancer treatment and diagnosis.^8,9^ Furthermore, NPs, such as palladium (Pd) and platinum (Pt), recognized for their distinctive properties arising from their size and structure, have been used to enhance the sensitivity of therapeutic agents.^10^ Although these NPs are widely used in chemotherapy drugs such as cisplatin due to their exceptional catalytic activity, they are equally recognized for their systemic toxicity and lack of specificity.^11^ To mitigate these difficulties and enhance their stability, biocompatibility, and cellular uptake,^12^ nanocomposite models of N-GQDs containing PEI doped with PdNPs have been synthesized. This strategy aims to increase the sensitivity of cellular targeting while simultaneously reducing unwanted side effects, making it a valuable tool for both imaging and drug delivery.^8,13,14^

Research indicates that GQDs can infiltrate cells and, engage with genetic material and proteins within the cell, leading to alterations in both the cell cytoplasm and nucleus, ultimately inducing cytotoxic effects.^15^ The cytoplasm-nucleus shuttling system observed in nanocomposites containing graphene positions makes them promising carriers for pharmaceutical applications.^16^ Exposure to graphene prompts an upsurge in intracellular reactive oxygen species (ROS), including superoxide generated by mitochondria within cells. This heightened ROS level triggers apoptosis through the release of pro-apoptotic molecules following a sequence of metabolic events from the mitochondria to the cytoplasm.^17,18^ It is noteworthy that normal cells also experience this toxic impact. Consequently, a prudent approach involves incorporating GQDs into a carrier system or potentially conjugating them with cancer cell-specific antibodies to mitigate cytotoxicity in healthy cells.^19^

Nanocomposite materials are among the most useful materials in today’s technology, as excellent properties can be obtained by combining two or more nanomaterials. Graphene-based nanocomposites possess excellent mechanical, electrical, thermal, optical, and chemical properties. These materials have potential applications in high-performance transistors, biomedical systems, sensors, and solar cells. As a result, combining graphene with inorganic materials such as metals has been the focus of research in recent years because of their multifunctionality.^20^

**Scheme 1:**
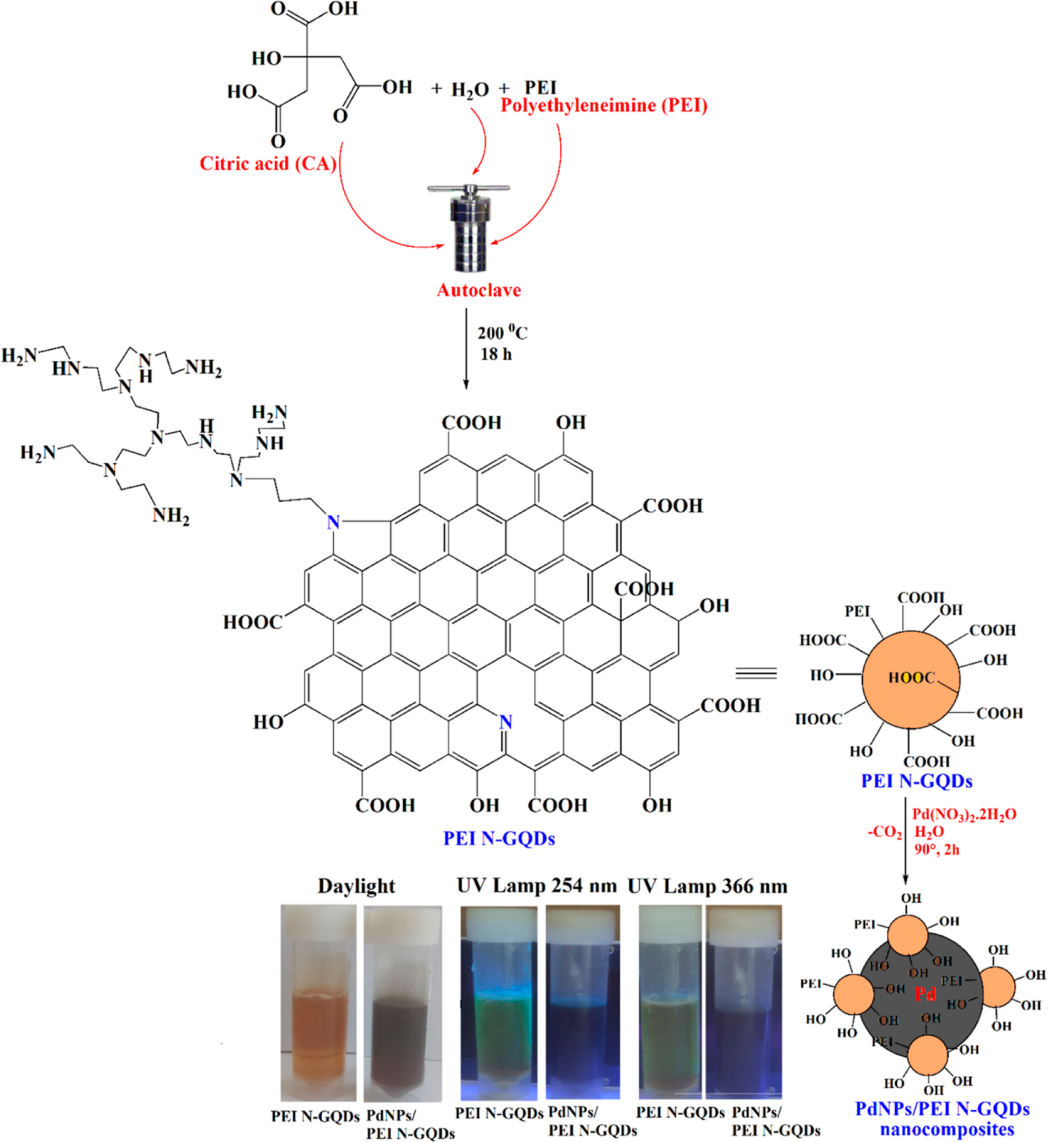
Synthesis of PEI N-Doped GQDs and PdNPs/PEI N-Doped GQD Nanocomposites

In this study, PEI-functionalized N-GQDs were synthesized primarily by the green method. Then, PdNPs/PEI N-GQD nanocomposites were prepared using the synthesized PEI N-GQDs as reducing and stabilizing agents for PdNPs in aqueous media (Scheme 1). The versatile effects of the characterized materials on OVCAR3 ovarian cancer cells were investigated, and Density Functional Theory (DFT) calculations. We also believe that studies on ovarian cancer cells, which continue to pose a significant threat to women’s health and are characterized by high mortality rates due to late diagnosis and limited treatment options, are important.^21^

## 2 Materials and Methods

### 2.1 Materials

Fourier transform infrared (FTIR) spectra, recorded in cm^–1^ units, were obtained using a PerkinElmer BX II spectrometer with KBr discs, while ultraviolet–visible (UV–vis) spectra were recorded with a PG Instruments T+80 UV–visible spectrometer. The morphological characterization of PEI N-GQDs and PdNPs/PEI N-GQDs nanocomposites was conducted by transmission electron microscopy (TEM) using an FEI Technai G2 STwin instrument at 200 kV. To prevent aggregation, nanocomposites in aqueous suspensions were treated in an ultrasonic bath before deposition. Nanostructures (10 μL) were placed on Formvar/carbon-coated 200-mesh copper grids and air-dried. The elemental compositions of PEI N-GQDs and PdNPs/PEI N-GQDs nanocomposites were analyzed using energy-dispersive X-ray photoelectron spectroscopy (XPS) with a Bruker AXs XFlash Detector 4010. X-ray diffraction (XRD) patterns were obtained on a Rigaku MiniFlex 600 X-ray diffractometer using Cu Kα radiation (λ = 1.54051 Å) in the 2θ range of 20–90° with a step size of 0.02 at 2 min^–1^ steps, operating at an accelerating voltage of 40 kV and a current of 15 mA. All chemicals, including citric acid (CA), Pd(NO_3_)_2_·2H_2_O, and polyethylenimine (PEI) (average *M*_w_ ≈ 25,000 by LS, average *M*_n_ ≈ 10,000 by GPC, branched), were commercially sourced from Sigma-Aldrich and used without further purification.

### 2.2 Synthesis

#### 2.2.1 Synthesis of PEI N-GQDs

PEI N-GQDs have been successfully synthesized using a hydrothermal method.^13,22^ Citric acid monohydrate (2.31 g, 10.00 mmol) and PEI (2.62 g, 1.00 mmol) were dissolved in 50 mL of deionized water, placed in a Teflon container, and placed in an autoclave. The autoclave was kept in an oven at 200 °C for 18 h. The suspension products in the autoclave cooled to room temperature were centrifuged at 12,000 rpm for 10 min, and the nanoparticles were collected. The collected PEI N-GQDs nanoparticles were washed twice with deionized water and once with ethanol. The obtained PEI N-GQDs were dried in a vacuum oven and stored in a desiccator.

#### 2.2.2 Synthesis of PdNPs/PEI N-GQDs Nanocomposites

PEI N-GQDs (0.0407 g) were added to a 250 mL round-bottom flask, and 50 mL of water was added. Then, 50 mL of solution of 0.0108 g of Pd(NO_3_)_2_·H_2_O was added to this mixture, and the mixture was heated in a water bath at 90 °C for 2 h to form PdNPs/PEI N-GQDs nanocomposites. After this time, the color of the solution changed from yellow to gray-black, and the palladium cation (Pd^2+^) was completely reduced to a palladium (Pd^0^) metal. The solution was filtered and the solid was washed twice with deionized water. The powdered PdNPs/PEI N-GQDs nanocomposites were dried in a vacuum oven and stored in a desiccator for further use.

### 2.3 DFT Method

**Scheme 2:**
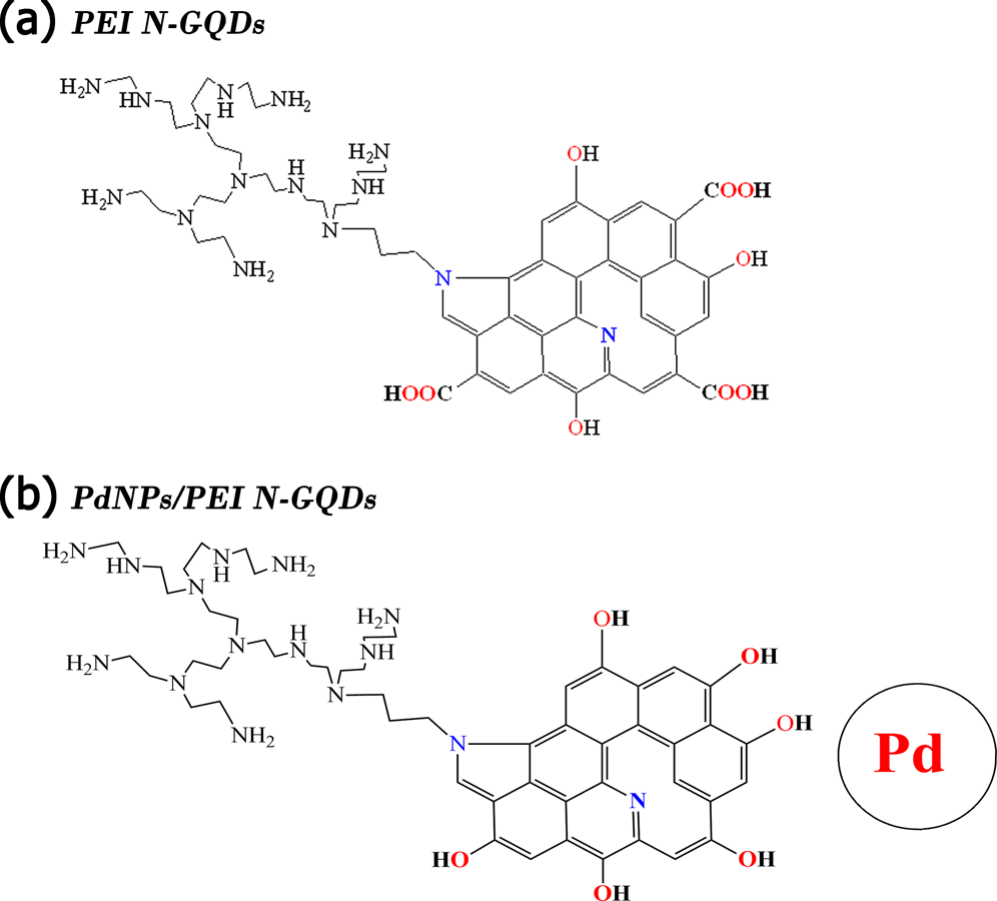
Theoretical Model of (a) PEI N-Doped GQDs and (b) PdNPs/PEI N-Doped GQDs

In this section, a comprehensive investigation of PdNPs/PEI N-GQDs nanocomposites, including the reference source PEI N-GQDs from our previous work,^13^ has been performed using the DFT method. The main objective of this research is to evaluate the sensitivity and selectivity of these materials by analyzing their electronic properties. Here, it will also be crucial to consider several important calculations, such as geometry optimization, electronic structure analysis, charge distribution and transfer, and vibration analysis, to obtain information about the electronic, structural, thermal, and energetic properties of these materials to support the testing of the effect of these materials on cancer cells. First, our computational system faced limitations in performance and efficiency due to the significant dimensions of the experimental nanocomposites. As a result, it could not effectively manage the required calculations. Second, the use of X-ray diffraction to determine the molecular geometrical structure was not possible because of certain limitations. To overcome these difficulties and proceed with the analysis of electronic properties using the DFT method, we needed to simplify the models while maintaining their similarity. This task involved the use of Gaussview 5.0 visualization software^23^ to create a novel molecular structure. Specifically, we sought to enhance the structure of PEI N-GQDs, as previously described in ref (13). Our objective was to render this enhanced PEI N-GQDs structure more compatible with further modifications. To achieve this, we embarked on a series of modifications. First, we meticulously introduced hydrogen (H), hydroxyl (OH), and carboxylic acid (COOH) groups to distinct edge points of the original PEI N-GQDs molecular framework. These groups were strategically incorporated to ensure improved compatibility and functionality within the structure. The next significant step in this process was the incorporation of PdNPs into the modified structure. These compounds were methodically integrated into the framework, resulting in the establishment of theoretical models for the PEI N-GQDs. This is illustrated in Scheme 2.

To find the most stable structural configuration of these theoretical models, they were optimized using the DFT/B3LYP method using the Gaussian 09W software package.^24^ The Los Alamos National Laboratory 2 double-ζ (Lanl2dz) basis set was applied in the ground state for accurate calculations.^25^ LanL2dz is a basis set developed for calculations of transition metals and is therefore useful in calculations of such quantum dots. Therefore, using the LanL2dz basis set when calculating the structural, thermodynamic, and spectroscopic properties of platinum group metals helps to obtain more accurate results.^26^ In addition, the FTIR spectra were recorded, which allows a detailed interpretation of molecular vibrations and provides valuable information about the structure, functional groups, and chemical behavior of the investigated nanocomposites. Furthermore, this basis set facilitated the determination of important properties, such as the highest occupied molecular orbital (HOMO), lowest unoccupied molecular orbital (LUMO), the molecular electrostatic potential (MEP), and HOMO–LUMO energy gap. A comprehensive analysis was carried out by creating density of state (DOS) plots using Gauss-Sum v3.0 software,^27^ which helped to visualize the electronic states of the investigated nanocomposites. In addition to DOS plots, theoretical data in the ultraviolet–visible (UV–vis) range were also generated using a computational technique called timedependent density functional theory,^28^ combined with the CAM-B3LYP method and the same basis set. This method, frequently utilized for predicting how molecules behave in UV and visible spectra,^29^ provided valuable insights into the electronic properties of nanocomposites alongside the DOS analysis. Furthermore, we performed an examination using the independent gradient model (IGM) via the Multiwfn software,^30^ a notably efficient method ideally suited for the investigation of complex systems, such as the PEI N-GQDs nanocomposite incorporating PdNPs.

### 2.4 Cell Culture

The human ovarian carcinoma cell line OVCAR-3 was obtained from the American Type Culture Collection (ATCC, Rockville, MD, USA). Cells were cultured in RPMI-1640 medium containing l-glutamine (Bio-Ind, USA), 20% fetal bovine serum (Bio-Ind, USA), 100 U/mL penicillin, and 100 mg/mL streptomycin (Gibco/USA) at 37 °C in 5% CO_2_ and 95% humidity. The culture medium was refreshed every 2 days. Cells were dissociated with 0.25% trypsin and passaged when the cells reached 75–85% confluence.

### 2.5 Cell Viability Assay

In 96-well plates, 1 × 10^4^ cells/well were seeded for 24 and 48 h, respectively, and the cells were exposed to varying concentrations (0.24, 0.49, 0.98, 1.95, 3.91, 7.81, 15.63, 31.25, 62.5, 125, 250, and 500 μg/μL) of PEI N-GQDs and PdNPs/PEI N-GQD nanocomposites. The 3-(4,5-dimethylthiazol-2-yl)-2,5-diphenyltetrazolium bromide (MTT) assay (Sigma, USA) was used to determine the cell viability. Following 24 and 48 h of treatment with each nanocomposite, the MTT test was used to determine cell proliferation. Subsequently, the cells were incubated for an additional 4 h at 37 °C with 5 mg/mL MTT solution. The culture medium containing MTT was removed, and formazan crystals were dissolved in 100 μL of isopropanol. Using a spectrophotometric plate reader (BioTek, USA) set at both 550 and 690 nm, the optical density of each experiment was determined. The percentage of cell viability in each treated group was computed on the basis of an acceptance of 100% for untreated cell viability. Distilled water served as both a negative control and a solvent for the nanocomposites in each group. Each nanocomposite and distilled water had a maximum concentration of 0.5%.

### 2.6 Annexin V/7-Aminoactinomycin (7-AAD) Staining

OVCAR-3 cells were placed in 24-well plates at a density of 1 × 10^5^ cells per well. Subsequently, each nanocomposite was applied to treat the cells for 48 h. Following the manufacturer’s guidelines, the cells were collected after 48 h, twice cleaned with PBS, centrifuged, and suspended in binding buffer (1× BB). In summary, equal amounts of PE-Annexin V (5 μL) and 7-AAD (5 μL) were added to a 100 μL cell suspension, which was then incubated for 15 min at room temperature (BD Biosciences, USA). Following incubation, 400 μL of 1× BB was added. Apoptosis was examined using flow cytometry (BD Accuri C6 Flow Cytometer).

### 2.7 Cell Cycle Analysis

OVCAR-3 cells (1 × 10^5^/well) were collected and fixed in 75% ice-cold ethanol (Merck, Germany) at 4 °C for 30 min following a 48 h culture in a medium containing each nanocomposite and distilled water. Subsequently, they were rinsed with 2× PBS. Following the washing process, the supernatant was eliminated and cells were treated with RNase (Sigma, USA) (50 μg/mL) for 15 min at 37 °C and propidium iodide (PI) (Sigma, USA) (50 μg/mL) for 15 min at 4 °C in the absence of light. The DNA content was measured using flow cytometry (BD Accuri C6 Flow Cytometer). To identify the cell cycle stage of individual cells, measurements of side scatter (SS) and forward scatter (FS) were made.

### 2.8 Statistics

Three replicates of each experiment were performed. The standard deviation (SD) was used to determine the mean of all experimental data. GraphPad Prism software was used to analyze the data. A two-way ANOVA test was used to assess the differences between the control and treatment groups. *p* < 0.05 was determined as a statistical significance.

## 3 Results and Discussion

### 3.1 Characterization of PEI N-GQDs and PdNPs/PEI N-GQDs Nanocomposites (FT-IR, UV–Vis, XRD, SEM, TEM, and XPS)

In the FTIR spectrum of PEI N-GQDs, the frequencies of the characteristic functional groups are ν[OH + NH_2_ + NH + COOH]; 3433 s-br, νC–H; 2931 s, νC=O: 1702–1698 s-br, νC=N_pyridinic_; 1654 s, νC=C; 1557 s, νC–N; and 1440 m, νC–O; observed at 1302 cm^–1^. In the spectrum, the OH + NH_2_ + NH + COOH peaks overlapped. In addition, the characteristic C=O (carboxylic acid) and C=N (pyridinic) vibrations in PEI N-GQDs were observed at different frequencies. In PdNPs/PEI N-GQD nanocomposites, OH, NH_2_, NH, COOH, C–H, COO, C=N, C=C, C–N, and C–O vibration bands are observed at 3850–3740, 3542–3472, 3410, 3226, 2954–2918–2861, 1736, 1635, 1555, 1454, and 1397 cm^–1^, respectively. In the FTIR spectrum, C=C and C–H bending vibrations were observed at 772–568 cm^–1^ in PEI N-GQDs and 816–785–616 cm^–1^ in PdNPs/PEI N-GQDs composites, respectively. The FTIR spectra of the investigated PEI N-GQDs and PdNPs/PEI N-GQD nanocomposites are illustrated in Figure S1.

When the vibrations in the FTIR spectra of PdNPs/PEI N-GQDs nanocomposites are compared with those in the spectrum of pure PEI N-GQDs, the vibrations of all functional groups in the metal nanocomposites are shifted to higher frequencies. From these results, it can be seen that GQDs are oxidized, while metals are reduced.

**Figure 1:**
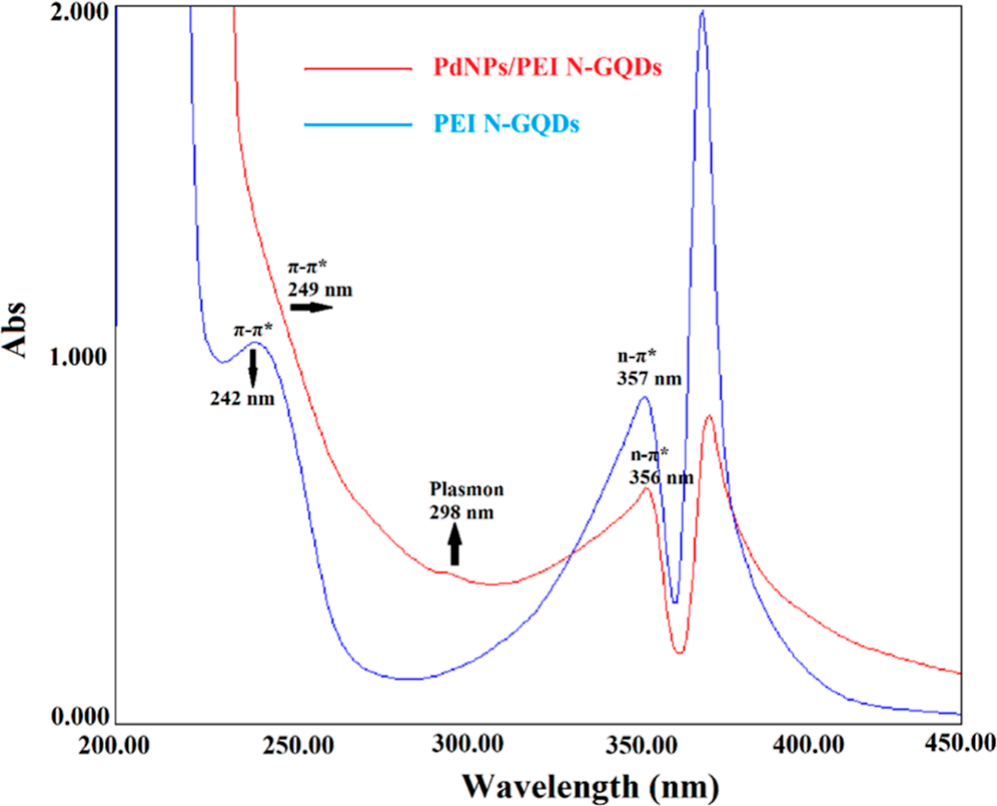
Absorption spectra of suspensions of PEI N-GQDs and PdNPs/PEI N-GQDs nanocomposites in water.

The absorption spectra of suspensions of PEI N-GQDs and PdNPs/PEI N-GQDs nanocomposites in water were measured (Figure 1). In the UV–vis spectrum of PEI N-GQDs, two bands assigned to π–π* transitions of C=C at 242 nm and *n*–π* transitions of C=N and C=O at 357 nm are observed (Figure 1). In PdNPs/PEI N-GQDs nanocomposites, π–π* and *n*–π* transitions were observed at 249 and 251 nm. Furthermore, the shoulder at 298 nm was assigned to the plasmon resonance in PdNPs/PEI N-GQD nanocomposites (Figure 1).

**Figure 2:**
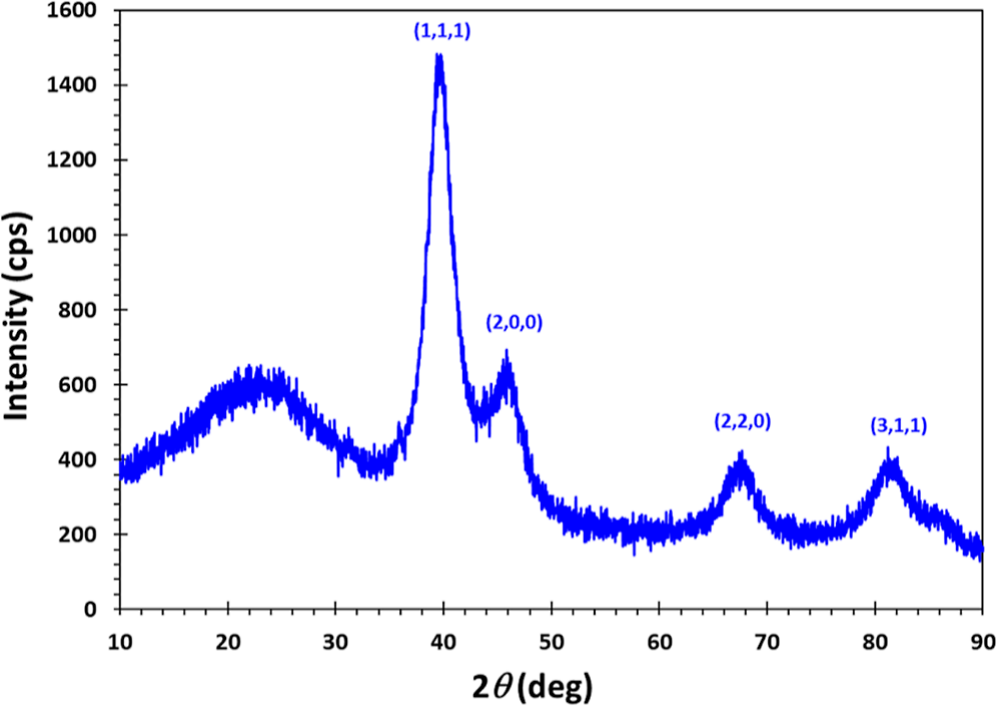
XRD pattern of the PdNPs/PEI N-GQDs nanocomposite.

The XRD pattern of the PdNPs/PEI N-GQD nanocomposites is shown in Figure 2. The curves obtained for PdNPs/PEI N-GQDs show a broad peak centered at approximately 2θ = 24.55°. The broad peak centered around 24.5° in PdNPs/PEI N-GQDs belongs to pure graphene samples and graphene nanosheets.^31^ The XRD profiles of N-free GQDs and N-containing N-GQDs were studied ^3^. A broader diffraction peak at approximately 25° was observed in N-GQDs, which is 1.5° higher than that of the normal graphene (about 23.5°). Again, the interlayer spacing in N-GQDs was found to be approximately 0.34 nm compared to the original graphene investigated by XRD (about 0.37 nm). The reduction in the interlayer spacing in N-GQDs was attributed to efficient π–π stacking and possible hydrogen bond formation between O-containing functional groups surrounding the edges of the graphene layers in N-GQDs. Also, it is noteworthy that no diffraction is observed in the 2θ = 10° region in N-GQDs, which is characteristic of graphene oxides. In another study, the XRD pattern of N-GQDs showed a broad diffraction peak at 2θ = 21.06° corresponding to the crystal facet of graphene (002).^32^

Pd-graphene nanocomposites were synthesized and its XRD pattern were analyzed.^33^ According to the XRD patterns of graphene and Pd composites, typical peaks (002) and (100) of graphene were observed at about 2θ = 26 and 43°, respectively, which are indexed to the plane reflections of graphite from graphene characteristic peaks (002) and (100).^33^ The intensity of the peaks in the free graphene compound decreased for all metal–graphene composites, as the metal particles were dispersed on the graphene surface. For the Pd-graphene composite, peaks around 40.1, 46.6, and 68.1° were observed, which can be indexed by the characteristic peaks (111), (200), and (220) of the Pd crystalline structure.^33^

When the XRD pattern of our synthesized PdNPs/PEI N-GQDs is examined in Figure 2, the PdNPs/PEI N-GQDs nanocomposites, the characteristic peaks of Pd were observed at 39.82, 45.93, 67.14, and 81.01° indexed by (111), (200), (220), and (311).^33^ In this study, we calculated the thickness of the layers of PEI N-doped GQDs using the Bragg eq (2*d* sin θ = λ). According to calculations from the Bragg equation, the layer thickness was found to be 1.86 Å for the PdNPs/PEI N-GQDs nanocomposite. Furthermore, the average crystallite size of PEI N-GQDs, Pd metal was calculated using the Scherrer formula given below.^34^
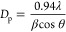
1where *D*_p_ = average crystallite size, β = line broadening in radians, θ = Bragg angle, and λ = X-ray wavelength.

According to the calculations from eq 1, in the PdNPs/PEI N-GQDs nanocomposite, the average size of PEI N-GQDs was 4.24 nm, and that of PdNPs was 12.62 nm. As a result, when comparing the XRD patterns of PEI N-GQDs and PdNPs/PEI N-GQDs nanocomposites that we synthesized, it becomes evident that the characteristic peak profiles and plane reflectance indexing for Pd are remarkably similar or closely aligned, while those of PEI N-GQDs differ significantly.

**Figure 3:**
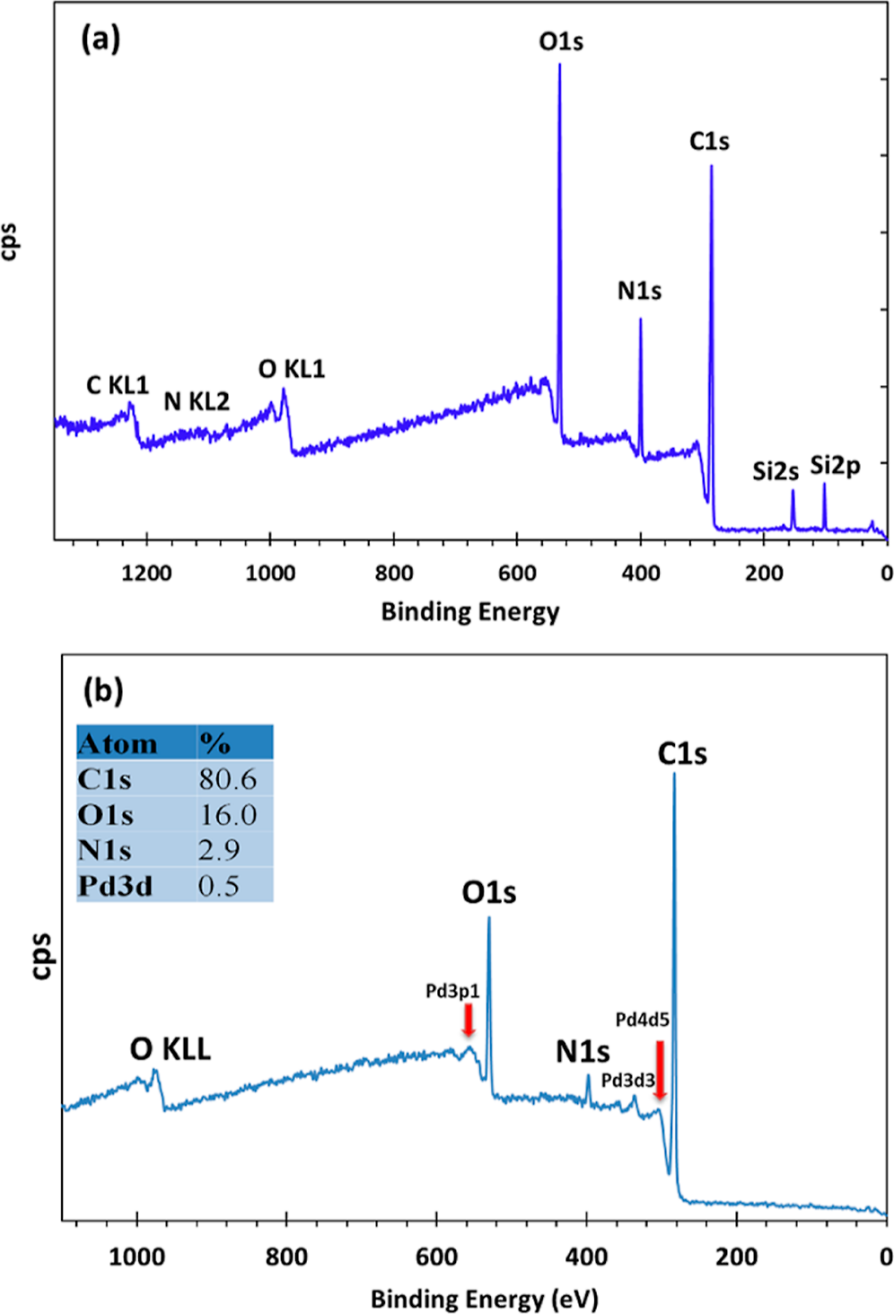
XPS spectra of (a) the carbon, nitrogen, and oxygen bond configurations of PEI N-GQDs and (b) the carbon, nitrogen, and oxygen with Pd bond configurations in the PdNPs/PEI N-GQDs nanocomposite.

The elemental compositions and bonding configurations of PEI N-GQDs and PdNPs/PEI N-GQDs nanocomposites were analyzed by XPS spectroscopy (Figure 3a,b). The carbon, nitrogen, and oxygen binding energies of PEI N-GQDs were found to be 284.58, 400.08, and 531.58 eV, respectively (Figure 3a). Furthermore, carbon, nitrogen, and oxygen auger peaks (CKL1, NKL2, and OKL1) are observed at 1229.08, 1108, and 980 eV in PEI N-GQDs. The spectra of carbon, nitrogen, oxygen, and Pd bond configurations in PdNPs/PEI N-GQDs nanocomposites are as given in Figure 3b. When Figure 3b is analyzed, the peaks are at 283.19 (C 1s), 398.64 (N 1s), 530.13 (O 1s), 558.76 (Pd 3p1), 336.55 (Pd 3d3), and 306.48 (Pd 4d5) eV, respectively. In addition, the oxygen auger peak (O KL1) is observed at 976.66 eV in the PdNPs/PEI N-GQDs nanocomposite.

In XPS, two different X-ray sources are used to distinguish auger peaks from photoelectron peaks. The auger peak represents the kinetic energy of an auger electron, which varies with the energy of the primary X-rays. Therefore, when the X-ray source is switched, the auger peak appears in the XPS spectrum, and the binding energy also shifts. When a photoelectron is emitted, the space (hole) vacated by the electron leaving can be occupied by an electron in the outer shells. There are two possibilities for this. In the first case, an outer shell electron is transferred to the hole by emitting a photon with a transferred energy difference. In the second case, an outer shell electron interacts with an electron in another shell and transfers this energy difference to the interacting (auger) electron. This electron is the auger electron and can be emitted if the transferred energy is higher than the binding energy. The difference determines the kinetic energy of the auger electron. Moreover, this energy does not depend on the energy by volume (*hv*) of the primary photon. According to the XPS results, it can be concluded that PdNPs/PEI N-GQDs nanocomposites were formed, and their structures were as given.

**Figure 4:**
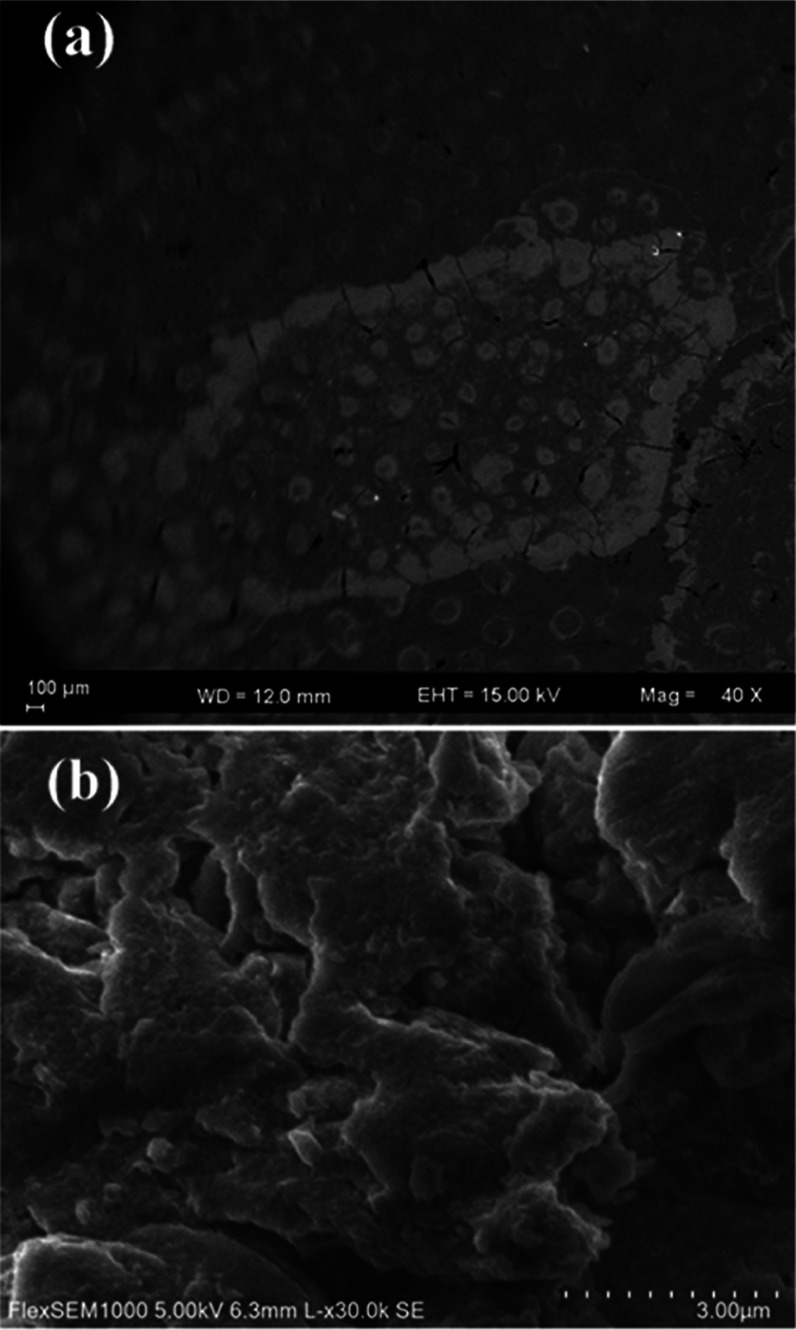
SEM images of (a) PEI N-GQDs and (b) PdNPs/PEI N-GQDs.

**Figure 5:**
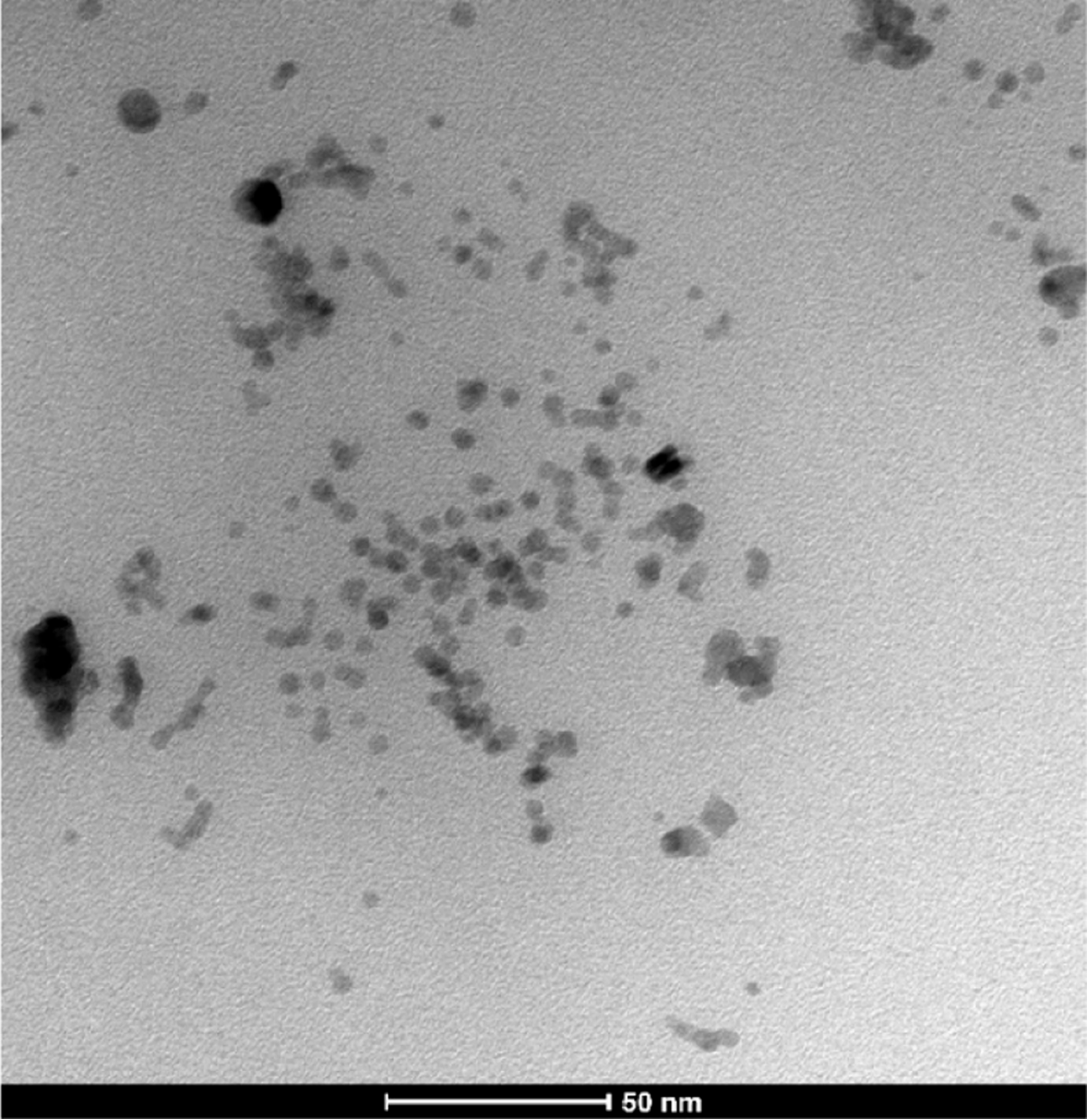
TEM image of PdNPs/PEI N-GQDs.

The morphology of PEI N-GQDs, along with the nanocomposites PdNPs/PEI N-GQDs, was scrutinized using SEM (Figure 4a,b) and TEM (Figure 5) analysis. The SEM image in Figure 4a shows that PEI N-GQDs have spherical particle sizes between 5 and 20 nm and do not form agglomerates. On the other hand, the PdNPs/PEI N-GQDs nanoparticles in Figure 4b are formed in relatively uniform layers due to regular growth and expansion.

The TEM image of PdNPs/PEI N-GQDs nanoparticles shows that Pd forms spherical and porous structures with an average size of 2–10 nm and does not form agglomerates (Figure 5). From the TEM images, it was observed that PEI N-doped GQDs retained their spherical porous structure and were randomly distributed between the metals, whereas Pd had spherical porous structures.

The particle sizes obtained from the XRD, SEM, and TEM results are quite consistent. According to the TEM results, the PdNPs/PEI N-GQDs nanoparticles showed a spherical form, while the SEM results of PEI N-GQDs nanoparticles showed a deformed spherical structure. Therefore, PdNPs/PEI N-GQDs nanoparticles with smaller sizes and spherical structures were more effective on cancer cells than PEI N-GQDs with a larger size and deformed spherical structure.

### 3.2 DFT Analysis

**Figure 6:**
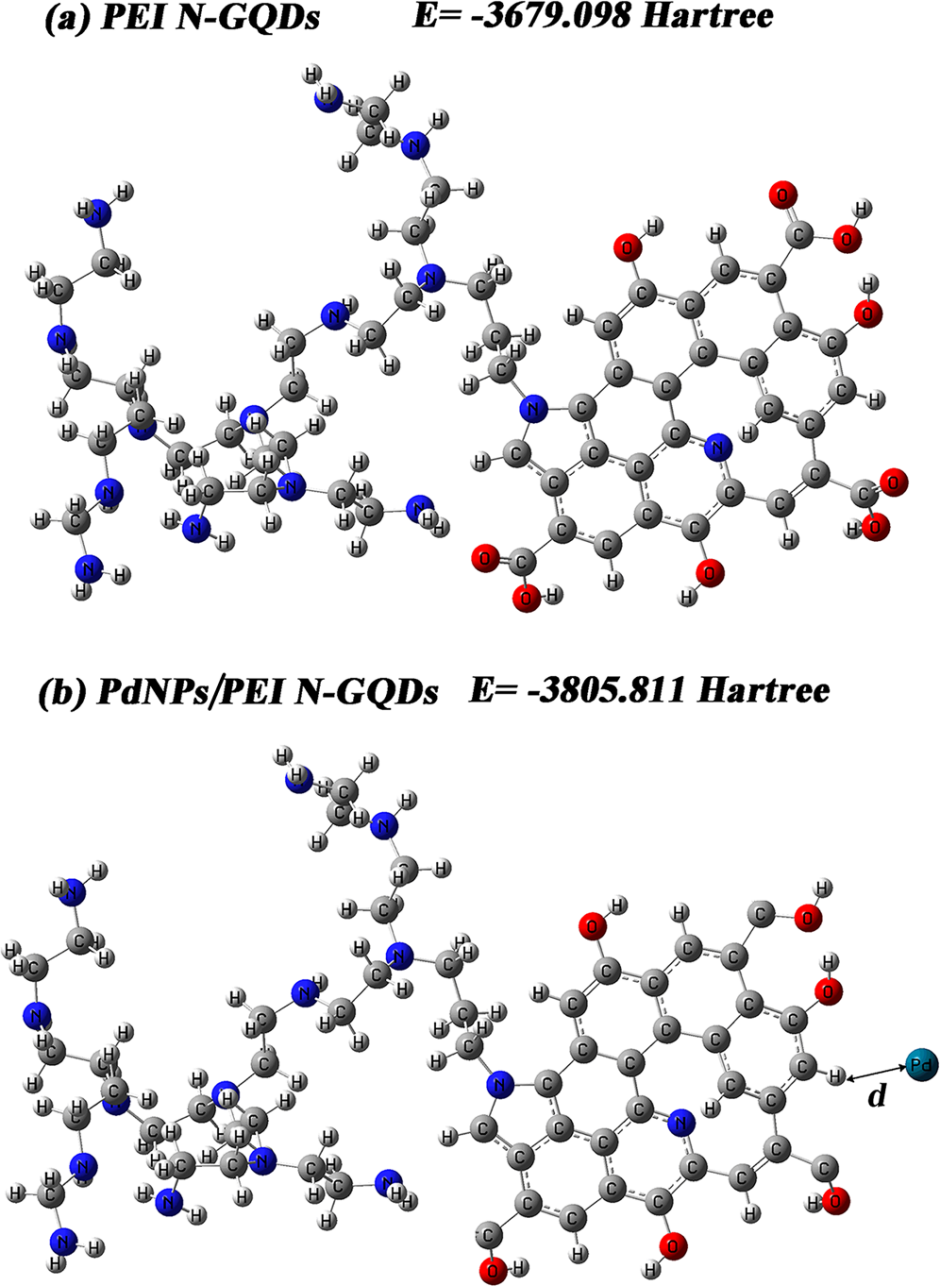
Optimized molecular structures of (a) PEI N-doped GQDs and (b) PdNPs/PEI N-doped GQDs nanocomposites.

To begin with, a geometric optimization calculation was executed to identify the nanocomposites’ minimum energy structure, which corresponds to their most stable configuration in the gas phase. In the gas phase, molecules have unrestricted movement, and intermolecular interactions are minimal compared to those in other phases. Therefore, the gas phase offers an advantageous environment for observing the fundamental energy levels of the molecular structures. Consequently, optimized molecular configurations in the gas phase serve as foundational starting points for numerous chemical and physical analyses. This calculation resulted in the attainment of a stable structure, representing the lowest energy state, achieved by optimizing the positions of all atoms within the molecule, as depicted in Figure 6a,b. The optimized PEI N-GQDs and PdNPs/PEI N-GQDs display the lowest energy levels, measuring at −3679.098 and −3805.81, respectively. The PdNPs/PEI N-GQDs nanocomposite stands out as a potential candidate for the highest stability, a critical factor in optimizing binding affinity and enhancing drug design due to its lowest energy level. Given that the nanomaterials under investigation were synthesized experimentally in a water medium and the analyses were carried out accordingly, the optimized structures of the nanocomposites will be employed in a water solvent in our forthcoming theoretical calculations to facilitate a meaningful comparison. Our overarching objective is to enhance cancer treatment methodologies and contribute to the development of more efficient, precise, and fewer side-effect-inducing therapies. To achieve this goal, we conducted assessments of the thermal and electronic properties of nanomaterials under scrutiny. The results are detailed in Table 1.

**Table 1:** Electronic and Thermal Properties of Nanocomposites Investigated at *T* = 298.15 K in Water Media

compounds	dipole (Debye)	polarizability (a.u.)	*E*_thermal_ (eV)	heat capacity (cal/mol K)	entropy (cal/mol K)
PEI N-doped GQDs	6.92	733.53	897.62	306.14	443.19
PdNPs/PEI N-doped GQDs	6.62	800.84	898.63	310.23	460.78

As shown in Table 1, Pd nanoparticles, which are renowned for their catalytic traits involving electron exchange processes, possess the capacity to either contribute or accept electrons when interacting with the nanocomposite. This interaction initiates a redistribution of charge within the system, which, in this context, reduces the overall dipole moment of the nanocomposite by counterbalancing any initial charge separation present in the PEI N-GQDs. With regard to polarizability, the inclusion of PdNPs into the nanocomposite leads to the infusion of fresh electron density, enhancing the overall polarizability of the system. Metals such as Pd exhibit substantial electron density due to their metallic characteristics, further increasing the nanocomposite’s polarizability. While the dipole moment values for PdNPs/PEI N-GQDs decrease relative to those for PEI N-GQDs, indicating a reduction in the separation between positive and negative charges within a molecule or material, there is also an observed increase in the polarizability values of these nanocomposites. This indicates an improved ability of the electron cloud to undergo distortion. A decrease in the dipole moment implies that the molecule or material becomes more evenly charged, which can result from changes in the molecular structure or electron distribution. Conversely, an increase in polarizability typically occurs when the electron cloud becomes more dispersed with electrons distributed over a larger volume. This can happen when the electron density is less concentrated around specific atoms or when there is an expansion in electron delocalization. In addition, when we analyzed the thermal properties of nanocomposites formed by doping PdNPs into the studied material and compared them with PEI N-GQDs, we did not observe much change in their heat energy at room temperature (*T* = 298.15 K), whereas a significant increase was observed in terms of heat capacity and entropy. Notably, the increase in heat capacity, a crucial factor in hyperthermia therapy requiring precise heating of nanoparticles to eliminate cancer cells, and the elevated entropy values indicating greater disorder and randomness within the system are particularly remarkable in the case of these nanocomposites. To put it simply, the heightened heat capacity implies that the nanocomposite can effectively absorb and retain a significant amount of thermal energy without experiencing a substantial temperature increase. However, it also means that nanocomposites with high entropy may have a reduced tendency to aggregate when in contact with cancer cells with high entropy as opposed to healthy cells.^35^ Consequently, this may enhance the stability of the nanocomposites during storage and release. Taking into account experimental assessments, such as toxicity and the ability to target cancer cells, these nanocomposites have emerged as a more promising choice for drug delivery and cancer therapy.

To further analyze the molecular vibrations, we also created an FT-IR spectrum, which provided us with crucial details regarding the molecule’s structure, functional groups, and chemical behavior. To confirm the precision of our structural assignment, we compared the anticipated spectrum derived from the proposed structure with the experimental data. Upon comparing the theoretical FTIR spectra of the PdNPs/PEI N-GQDs nanocomposite to the pristine PEI N-GQDs spectrum, we observed a slight upward shift in the vibrations of all functional groups in the metal nanocomposites, as in the experimental data (refer to Figure S2). In PdNPs/PEI N-GQD nanocomposites, vibration bands corresponding to OH + NH_2_ + NH + COOH appear between 3751 and 3391 cm^–1^, while C–H, COO, C=N, C=C, C–N, and C–O vibration bands are observed at 2956, 1716, 1625, 1555, 1473, and 1388 cm^–1^, respectively. The close alignment between the experimental and theoretical FTIR findings not only validates our theoretical model but also rigorously evaluates the precision and reliability of subsequent computational predictions, as demonstrated in Figure S2.

**Figure 7:**
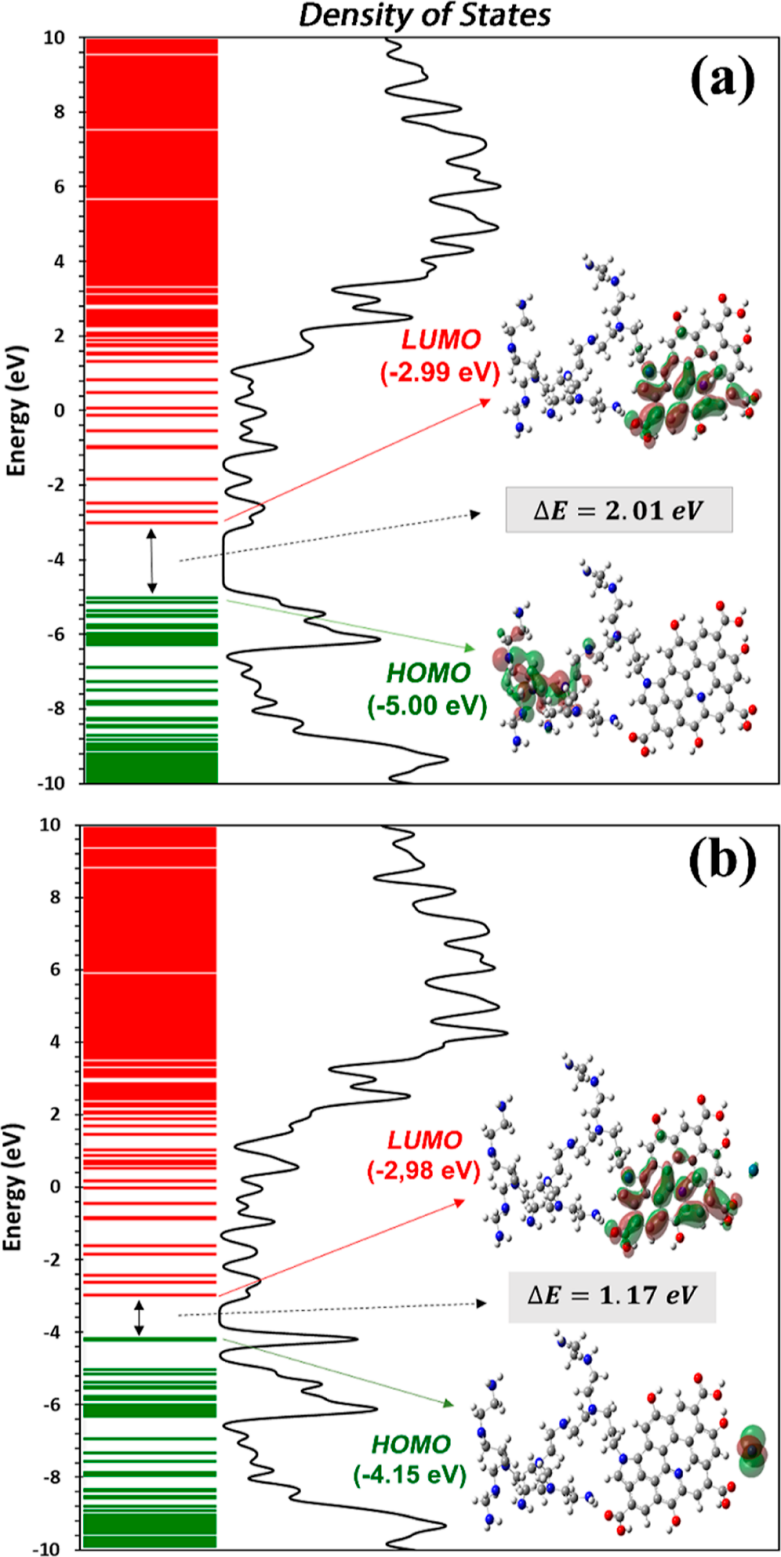
DOS spectra with HOMO and LUMO visualization and energy gap (Δ*E*) of the (a) PEI N-doped GQDs and (b) PdNPs/PEI N-doped GQDs nanocomposites.

Frontier molecular orbitals (FMOs) consist of two fundamental molecular orbitals known as the HOMO and the LUMO. As can be seen in Figure 7, the HOMO is typically occupied by electrons and possesses the highest energy level (green lines), functioning as an electron donor. Conversely, the LUMO remains unoccupied and holds the lowest energy level (red lines), serving as an electron acceptor.^36^ The energy difference between the HOMO and LUMO, termed the HOMO–LUMO gap, is of great significance. It provides insights into a molecule’s reactivity.^37^ In drug development, a smaller HOMO–LUMO gap often signifies higher reactivity, which can be advantageous for targeting specific biological molecules or pathways within cancer cells.^38^ Essentially, this method assists in forecasting a molecule’s likelihood to participate in chemical reactions. This strategy is based on the concept that chemical stability is linked to the processes of electron transfer. To be more precise, during chemical reactions, electrons are initially taken from the HOMO and transferred to the LUMO of the reacting substances.^39^

A molecule with a narrow HOMO–LUMO gap (Δ*E*) may be more inclined to participate in redox reactions,^40^ making it valuable for treatments relying on oxidative stress, such as certain cancer therapies. Furthermore, FMOs aid in evaluating the stability of drug molecules such as an anticancer agent. A stable molecule is less susceptible to undesirable reactions or degradation during drug delivery, ensuring that the therapeutic agent reaches its intended target intact. Additionally, understanding a molecule’s FMOs can provide insights into potential toxicities.^41^ Highly reactive molecules may inadvertently interact with unintended targets, leading to adverse effects. As given in Table 2 and also illustrated in Figure 7, the Δ*E* values calculated in a water medium were 2.01 and 1.17 eV for PEI N-GQDs and PdNPs/PEI N-GQDs nanocomposites, respectively. According to these results, the PdNPs/PEI N-GQDs nanocomposite appears to have an advantage. However, when the HOMO of this nanocomposite aligns well with the LUMO of the target molecule, it may exhibit a stronger binding affinity. This alignment is a critical factor in the design of potent drugs with high specificity for cancer therapy. Furthermore, interpretation of the HOMO and LUMO isosurfaces will provide additional valuable information about the electronic structure and reactivity of the molecules. The HOMO isosurface may be particularly important in drug delivery and cancer therapy to understand where electrons are concentrated within a molecule. As shown from the isosurfaces corresponding to HOMO and LUMO inserted in Figure 7, the distributions of the charge density in the investigated nanocomposites show delocalization in the red and green lobes on the HOMO–LUMO isosurfaces, which correspond to the positive and negative phases of the wave function, respectively, while the localization in other areas is maintained. The red lobes with a high electron density in the HOMO are prone to nucleophilic attack. In drug design, this information will help predict where a molecule may interact with biological targets. For example, a drug with a high electron density at its HOMO in a particular region may form strong bonds with the target protein or DNA molecule. On the other hand, the LUMO isosurface represents the region of low electron density, indicating an electron-deficient area of the molecule. This is usually associated with electrophilic regions. The LUMO isosurface can provide information about regions of a molecule that are susceptible to nucleophilic attack by biological molecules. The electronic properties represented by the HOMO and LUMO isosurfaces can influence the stability and potential toxicity of a molecule. Reactive sites in LUMO can lead to unwanted side reactions or toxicities when they interact with healthy cells or biomolecules. Figure 7 also shows the DOS spectra, a computational technique that provides valuable information about the electronic properties. The peaks in the DOS spectrum indicate a higher density of electronic states at those particular energy levels. In other words, there are more electrons or electronic states at these energy levels than other energy levels. This probably corresponds to certain electronic states in the nanocomposite that can interact with biological systems. Therefore, to elaborate further, peaks in the HOMOs may indicate electrons available for potential chemical interactions with external agents such as cancer cells or therapeutic drugs, while, conversely, peaks in the LUMOs may indicate the presence of electron vacancies that can enter redox reactions. Although the DOS plots for the investigated nanocomposites show remarkable similarity, we can conclude that the Pd atom doped in PEI N-GQDs significantly increases the density of states of the peaks at HOMOs and LUMOs compared with the other nanocomposites. This means that it can enter into chemical interactions more easily. It is important to compare the results of DOS analysis with experimental data derived from biological assays. We can state that the relationship between the electronic properties of the PdNPs/PEI N-GQDs nanocomposite and its effect on ovarian cancer cells, the specific energy levels or states identified in the DOS analysis, support the observed biological results such as cytotoxicity and apoptosis.

**Table 2:** FMOs Properties for the Investigated Nanocomposites in Water Media

FMOs properties|compound	PEI N-doped GQDs	PdNPs/PEI N-doped GQDs
*E*_HOMO_ (eV)	–5.00	–4.15
*E*_LUMO_ (eV)	–2.99	–2.98
Δ*E* = |*E*_HOMO_ – *E*_LUMO_| (eV)	2.01	1.17
ionization potential IP = −HOMO (eV)	5.00	4.15
electron affinity EA = −LUMO (eV)	2.99	2.98
hardness 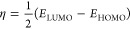 (eV)	1.01	0.59
electronegativity 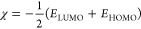 (eV)	4.00	3.57
electrophilicity ω = χ^2/2^η (eV)	8.02	3.72
softness σ = 1/ η(eV)	1.00	1.71

Furthermore, the study encompassed the computation of various parameters, including the ionization potential (IP), electron affinity (EA), hardness (η), electronegativity (χ), electrophilicity (ω), and softness (σ), with their corresponding values detailed in Table 2. Notably, *E*_HOMO_ describes the (IP), signifying electron-donating sites, whereas *E*_LUMO_ describes electron affinity (EA), representing electron-accepting sites.^42^

The I*P* values for PEI N-GQDs and PdNPs/PEI N-GQDs nanocomposites were calculated as 5.00 eV (EA: 2.99 eV) and 4.15 eV (EA: 2.98 eV), respectively. The lower EA of PdNPs/PEI N-GQDs indicates a weaker attraction to electrons, reducing their readiness to accept electrons and form anions. Conversely, its lower IP suggests a lower energy barrier for electron donation, making it more likely to donate electrons and form cations.^43^

The study also derived the parameters η, χ, ω, and σ,^42^ as presented in Table 2. These concepts are instrumental in understanding chemical systems. Soft compounds possess a narrower energy gap, whereas hard compounds have a wider energy gap. Based on these findings, PdNPs/PEI NGQDs appear to possess a notable capacity for polarization, suggesting its readiness to undergo polarization when interacting with other substances.

χ characterizes a compound’s electron-withdrawing capacity, while ω indicates its ability to donate charge. PdNPs/PEI NGQDs, compared to other nanocomposite, with its low electronegativity, are more inclined to donate electrons, serving as an electron source in chemical reactions. When described as electrophilic, it signifies that the nanocomposite readily accepts electrons and engages in electrophilic reactions by interacting with electron-rich sites.

**Figure 8:**
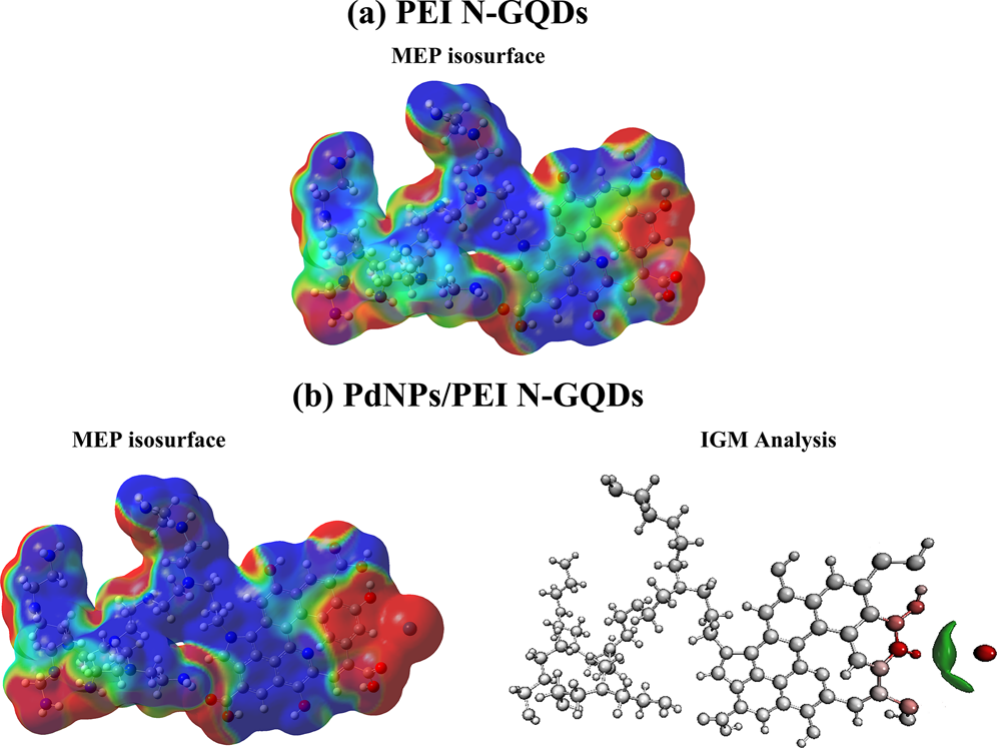
(a) MEP isosurface of PEI N-doped GQDs and (b) MEP isosurface and IGM analysis of PdNPs/PEI N-GQDs nanocomposites.

A different approach to pinpointing locations within a molecule that are prone to being targeted by electrophilic and nucleophilic reactions is through examination of the MEP. This method offers a different perspective on identifying sites within a compound that are susceptible to attacks by both electrophiles and nucleophiles. It relies on assessing the distribution of electric charge across the molecule, and the variation in this charge distribution helps to highlight areas that are more likely to attract electrophilic species (which seek electrons) and nucleophilic species (which have excess electrons). As depicted in Figure 8a,b, this approach uses color gradients on MEP surfaces to signify different reactivity regions. Negative electrostatic potential values linked to electrophilic reactivity are represented by red and yellow areas, whereas the blue areas indicate nucleophilic reactivity. The green region indicates a neutral potential. The MEP surfaces exhibit a gradient of colors, ranging from −0.01 atomic units (red) to 0.01 atomic units (blue) for all nanocomposites. The MEP surfaces reveal that the red areas contain significant electron concentrations, indicating electron-attracting reactive sites known as electrophiles. Conversely, the blue regions exhibit higher positivity, signifying the presence of sites that donate electrons, referred to as nucleophiles. However, it is important to note that “higher positivity” does not mean a lack of electrons; it means a surplus of electrons compared to the rest of the nanocomposite.

Within Pd-embedded N-GQD nanocomposites, there is a specific region where electrons are abundant. This region is densely populated (red region) only around PdNPs, which are one of the nanoparticles in the nanocomposite. The presence of extra electrons around the Pd atom indicates that a certain chemical interaction has occurred. This electron-rich region around the Pd atom is also a result of the chemical interaction between the Pd atom and a carboxyl group. The carboxyl group (−COOH) is a functional group containing a carbon atom doubly bonded to an oxygen atom and singly bonded to a hydroxyl (−OH) group. This interaction is described as electrophilic attraction. In this case, it means that the PdNPs attract electrons from the hydroxyl group and interacts. This information plays a pivotal role in comprehending the dynamics of molecular interactions within chemical reactions. Electrophiles and nucleophiles are drawn together to harmonize their electron distributions, thereby facilitating the creation of fresh chemical bonds. In fields such as drug design and the anticipation of chemical reactivity, a profound understanding of these regions empowers scientists to customize molecules for precise applications such as targeted drug delivery or cancer treatment by forecasting their behavior in interactions with other molecules within biological systems.

During this research, we also carried out an investigation employing IGM, a remarkably efficient methodology designed for the in-depth analysis of complex systems, specifically focusing on the composite material composed of N-GQDs incorporated with either Pd atom, as exemplified by the PEI N-GQDs nanocomposite (Figure 8b). Within this analysis, a noticeable van der Waals (vdW) interaction zone, particularly the π–π stacking region, is delineated by the green isosurface, with atoms displaying a deeper red hue signifying their greater contribution to this interaction. In this context, it becomes evident that adjacent –H atoms in the benzene ring and –O atoms within the –COOH and –OH groups (characterized by larger isosurfaces) engage in a π–π stacking interaction with PdNPs, thus categorized the interaction primarily as a vdW interaction. In drug delivery systems, such as nanoparticles, vdW interactions can influence the stability and loading capacity of drug carriers.^44^ Surface functionalization of nanoparticles with molecules that have complementary vdW interactions with drug molecules can enhance drug loading and controlled release.

In addition, we conducted a theoretical analysis of the UV–vis spectra for the compounds when they were dissolved in water-based media. This comprehensive examination delved into various spectroscopic properties associated with electronic transitions, encompassing parameters such as absorption wavelengths, excitation energies, and significant transitions. All of these were taken into consideration, including the highest oscillator strength,^45^ meticulously cataloged in Table 3.

**Table 3:** Optical Properties of PEI N-GQD**s** and PdNPs/PEI N-GQD**s** Nanocomposites in Water Media

	experimental			theoretical		
compounds	λ_abs_ (nm)	λ_abs_ (nm)	excitation energy (eV)	oscillator strength	major transitions	
PEI N-GQDs	357	339	3.66	0.95	H – 3 → L + 2	(50%)
	242	248	5.00	0.34	H – 20 → LUMO	(25%)
					H – 17 → LUMO	(11%)
					H – 1 → L + 6	(13%)
PdNPs/PEI N-GQDs	356	347	3.58	0.10	H – 12 → L + 1	(43%)
					H – 8 → L + 1	(12%)
	298	290	4.27	0.28	H – 21 → LUMO	(31%)
					H – 8 → L + 3	(12%)
	249	244	5.08	0.20	H – 25 → LUMO	(20%)

As can be easily seen in Table 3, with respect to the compound PEI N-GQDs, we identified three major transitions at 248 nm and one major transition at 339 nm, each associated with excitation energies of 5.00 and 3.66 eV, respectively. These transitions include electronic transitions from H – 20 to LUMO, H – 17 to LUMO, H – 1 to L + 6, and H – 3 to L + 2, contributing 25, 11, 13, and 50%, respectively. Similarly, the PdNPs/PEI N-GQDs compound exhibited peak absorbance at 244, 290, and 347 nm with corresponding excitation energies of 5.08, 4.27, and 3.58 eV. At 244 nm, it contains a single major electronic transition from H – 25 to LUMO with 20% contribution. At 290 nm, two major electronic transitions were detected from H – 8 to L + 3 and H – 21 to LUMO with contributions of 12 and 31%, respectively, while at 347 nm, two major electronic transitions were calculated from H – 8 to L + 1 and H – 12 to L + 1 with contributions of 12 and 43%, respectively. It is noteworthy that the UV–vis results closely align with the experimental data presented in Table 3.

**Figure 9:**
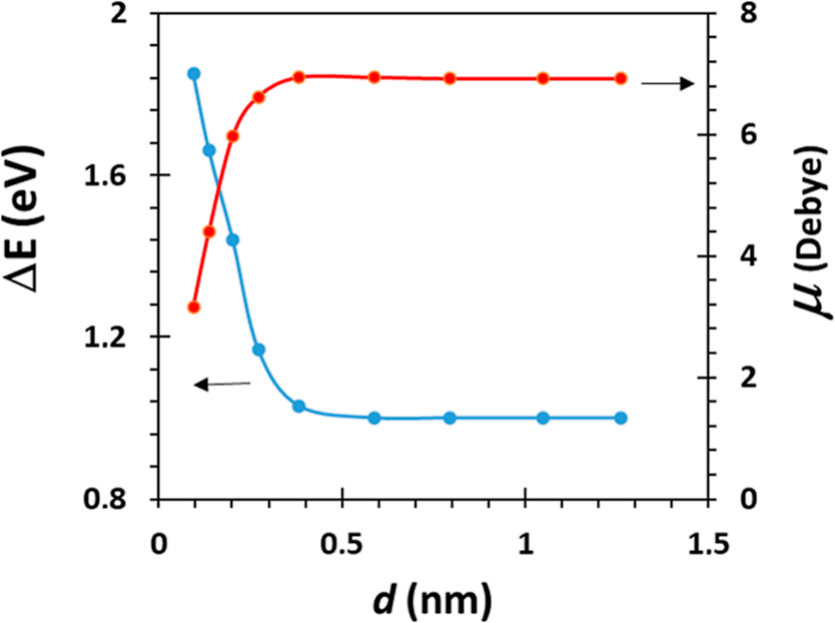
Variation of Δ*E* and μ concerning the position of the Pd atom (*d*) in the PdNPs/PEI N-GQDs nanocomposite.

So far, the calculations have been conducted based on a specific position of the PdNPs within the PdNPs/PEI N-GQDs nanocomposite. Exploring the influence of the Pd atom’s position on the electronic structure of PEI N-GQDs reveals significant changes in the dipole moment (μ) and the HOMO–LUMO energy gap (Δ*E*) relative to the distance between the nanocomposite and the Pd atom (*d*, as illustrated in Scheme 2). The decreasing energy gap with an increasing distance indicates a noticeable interaction between the Pd atom and the electronic states of the nanocomposite (Figure 9).

The identification of a specific point where the HOMO–LUMO energy gap remains consistent unveils a critical threshold. The observed stability beyond a certain distance suggests the presence of an optimal configuration or interaction distance that maintains the stability of the electronic structure. Additionally, the documented increase in the dipole moment (μ) up to a particular distance threshold indicates a dynamic interaction between PdNPs and the nanocomposite, potentially influencing the charge distribution within the system (Figure 9).

The sustained rise in the dipole moment beyond the specified threshold distance suggests that the nanocomposite has attained a relatively stable configuration concerning dipole properties, possibly indicating a uniformly distributed charge. These findings collectively contribute to a comprehensive understanding of the intricate interplay between PdNPs and the electronic and structural characteristics of the nanocomposite.

### 3.3 Effect of PEI N-GQDs and PdNPs/PEI N-GQDs Nanocomposites on Cellular Viability

Cell viability tests are frequently used methods to determine whether the molecules to be tested have an effect on the proliferation of cells or to show whether they cause cytotoxic effects in the cells and eventually cell death.^46^ There are many cell viability tests available, and in this study, we performed MTT analysis to evaluate the in vitro cell viability of PEI N-GQDs containing Pd nanoparticles on the OVCAR-3 cells.

**Figure 10:**
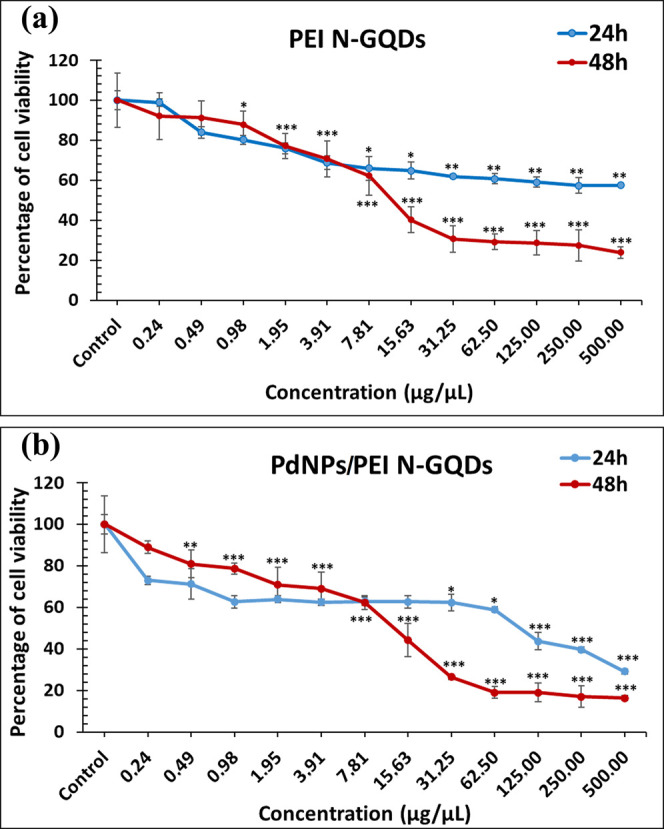
Effects of nanocomposites (0.24–500 μg/μL μM) (a) PEI N-doped GQDs and (b) PdNPs/PEI N-doped GQDs on cell viability of OVCAR-3 cells for 24 and 48 h. (*p < 0.05, **p < 0.001 and ***p < 0.0005 shows significant differences from the control group).

The cytotoxic effect of PEI N-GQDs and PdNPs/PEI N-GQDs on OVCAR-3 cell was evaluated by calculating IC_50_ from cell growth curves. The results are presented in Figure 10 and Table 4.

**Table 4:** IC_50_ Values of Nanocomposites in OVCAR-3 Cell Line

nanocomposites	IC_50_ (μg/μL) ± SD.
PEI N-GQDs	16.52 ± 1.69
PdNPs/PEI N-GQDs	13.76 ± 4.48

Each of the 12 distinct doses of nanocomposite, which were serially diluted by half, was given to OVCAR-3 cells at intervals of 0.24 and 500 μg/μL. Accordingly, compared with the untreated control group, the IC_50_ values of PEI N-GQDs and PdNPs/PEI N-GQD nanocomposites were found to be 16.52 ± 1.69 μg/μL (Figure 10a) and 13.76 ± 4.48 μg/μL (Figure 10b), respectively, and this result was statistically significant (Table 4) (*p* < 0.0005).

Zhang et al.^16^ emphasized that nanocomposites containing graphene can be used as carriers for drugs and genes due to the cytoplasm-nucleus shuttle system; therefore, GQDs are good candidates for pharmaceutical applications because of their physicochemical properties. Jiang et al.^15^ reported that GQDs can enter the cell, pass into the nucleus, and thus interact with both proteins and genetic material in the cell. They revealed that this interaction may ultimately cause a change in the gene expression level of the cell, disrupting the cytoplasm and nuclear morphology of the cell and reducing the cell viability. However, Wang et al.^19^ proposed that normal cells were also affected by this toxicity and suggested that GQDs could be put into a carrier system and conjugated with cancer cell-specific antibodies. Therefore, they produced N-GQDs.

We evaluated N-GQDs’ ability to bind DNA, support cell viability, and have antimicrobial and antioxidant properties in a different study that we conducted in 2019. The findings demonstrated that N-GQDs could bind to DNA through electrostatic and intercalation pathways; GQD-containing formulations were able to enter cells and reduce the number of cancer cells as well as the amount of EpHA2, which are highly expressed in lung cancer cells; high concentrations of quantum dots damaged DNA in cancer cells and decreased the viability of the cells. The effect of N-GQDs on breast and fibroblast cell proliferation was statistically significant, while the effect on lung cancer cells did not make a difference. The cytotoxic effect did not change with time.^6^ In this study, the cytotoxic effects of PEI-functionalized N-GQDs containing Pd nanoparticles on ovarian cancer cell lines were examined for the first time. Accordingly, PEI N-GQDs and PdNPs/PEI N-GQDs nanocomposites showed statistically significant higher cytotoxic effects compared to the control at low doses. Moreover, Figure 10 shows that the cytotoxic effects are different for all two composites. As the time increased, we observed an increase in cytotoxicity.

### 3.4 Apoptotic Effects of PEI N-GQDs and PdNPs/PEI N-GQDs Nanocomposites on Ovarian Cancer Cells

One of the main objectives of cancer treatments, especially chemotherapy, is to induce apoptosis in cancer cells. In anticancer research, any material or agent that can specifically inhibit the growth of malignant cells by altering the aberrant signal transduction pathway implicated in the cell cycle and/or the apoptotic mechanism is regarded as a crucial chemotherapeutic tool.^47^ This study found that applying PEI N-GQDs and PdNPs/PEI N-GQDs, nanocomposites to ovarian cancer cells, caused them to enter the statistically significant apoptotic death pathway. Accordingly, the flow cytometry method was used to show the type of cell death caused by PEI N-GQDs and PdNPs/PEI N-GQDs nanocomposites in ovarian cancer cells, and the experiments were repeated three times.

**Figure 11:**
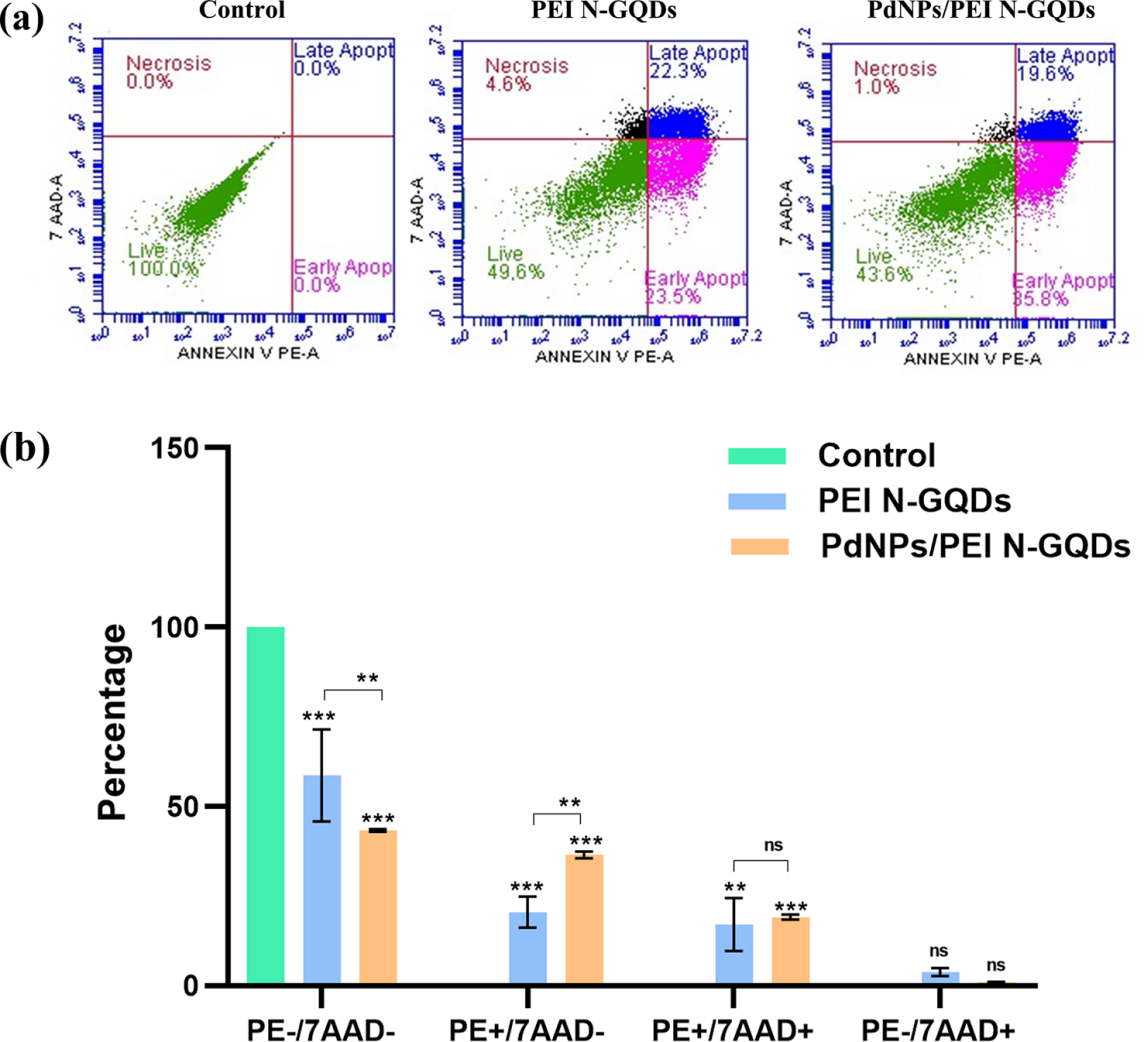
Nanocomposites induce apoptosis in vitro. Flow cytometric analysis of annexin V-PE/7-AAD-stained OVCAR-3 cells for 48 h. (a) Flow cytometry results are represented as dot plots. (b) Flow cytometry results of OVCAR-3 cells with/without nanocomposites were shown as bar graphs. Control; viability of distilled water (without nanocomposite)-treated cells was considered as 100%. Viable cells (PE–/7AAD−); early apoptotic cells (PE+/7AAD−), late apoptotic cells (PE+/7AAD+), and necrotic cells (PE–/7AAD+) (**p < 0.001 and ***p < 0.0005 and shows significant differences from the control and other groups).

In comparison to the cells in the untreated control group, the viability rate of ovarian cancer cells treated with PEI N-GQDs and PdNPs/PEI N-GQDs nanocomposites statistically decreased to, on average, 58.65% (58.65 ± 12.79) and 43.3% (43.30 ± 0.42) (*p* < 0.0005). The average percentage of cells going through early and late apoptosis rose to 20.45% (20.45 ± 4.31) and 17.1% (17.1 ± 7.35) in cells treated with PEI N-GQDs. The treated cells showed a significant early and late apoptosis rate compared with the control group (*p* < 0.0005 and *p* < 0.001, respectively). The percentage of cells undergoing early and late apoptosis rose statistically significantly to 36.5% (36.50 ± 0.99) and 19.1% (19.10 ± 0.71), respectively, in cells treated with PdNPs/PEI N-GQDs (*p* < 0.0005) (Figure 11a,b). The average rates of necrosis and/or dead cells in cells treated with PEI N-GQDs and PdNPs/PEI N-GQDs were 3.8% (3.80 ± 1.13) and 1.05% (1.05 ± 0.07), respectively, compared with the control group. However, these results were not statistically significant. Overall, the highest rate of apoptosis (55.6%) was observed in ovarian cancer cells treated with PdNPs/PEI N-GQDs and was followed by cells treated with PEI N-GQDs (37.55%) (Figure 11a,b).

A study by Qin et al.^48^ investigated that there were no significant structural changes in macrophages (phagocytic immune cells) treated with GQDs for a short time, whereas apoptotic death occurred in macrophages in long-term incubations with GQDs. Researchers have also reported that this death process is dose dependent and that the rate of apoptosis increases in macrophages as the dose increases. They concluded that GQDs induce cell apoptosis through a mitochondria-related pathway that activates the caspase family and apoptotic proteins.

Ou et al.^17^ reported that exposure to graphene increases the formation of intracellular ROS, including superoxide produced by mitochondria, and apoptosis is induced in cells after a series of metabolic events because of increased ROS in cells by releasing proapoptotic molecules from the mitochondria into the cytoplasm. Ramachandran et al.^18^ also supported this information in their study. According to them, human breast cancer cells to which N-GQDs/titanium dioxide nanocomposites (N-GQDs/TiO_2_) were applied under near-infrared (NIR) light produced ROS, and as a result, the cells started to process mitochondria-associated apoptotic cell death.

In this preliminary study, although we could not perform analyses such as ROS activity, mitochondria membrane potential testing, or gene and/or protein expression analyses to determine which apoptotic pathway is stimulated, we can say that our results support the few studies on the subject in the literature, and all three N-GQDs nanocomposites we used caused highly significant apoptosis in ovarian cancer cells. Furthermore, studies have demonstrated that the elevated ROS in Pd-treated cancer cells causes the cells to enter the mitochondria-dependent apoptotic pathway.^49^ According to this study, Pd-doped PEI N-GQDs triggered 15% more apoptosis than did undoped PEI N-GQDs. This means that when Pd is added, the apoptotic activity of undoped PEI N-GQDs increases even more.

### 3.5 PEI N-GQDs and PdNPs/PEI N-GQDs Nanocomposites Affect Cell Cycle Distribution in Ovarian Cancer Cells

It is commonly known that unchecked growth is the primary characteristic that distinguishes cancer. Numerous genetically defined Cyclin-dependent kinase inhibitors (CKIs) regulate cellular growth by allowing only healthy cells to advance through the G0/G1, S, and G2/M phases of the cell cycle; damaged cells undergo either irreversible damage repair or are directed to die through the process of apoptosis.^50^

**Figure 12:**
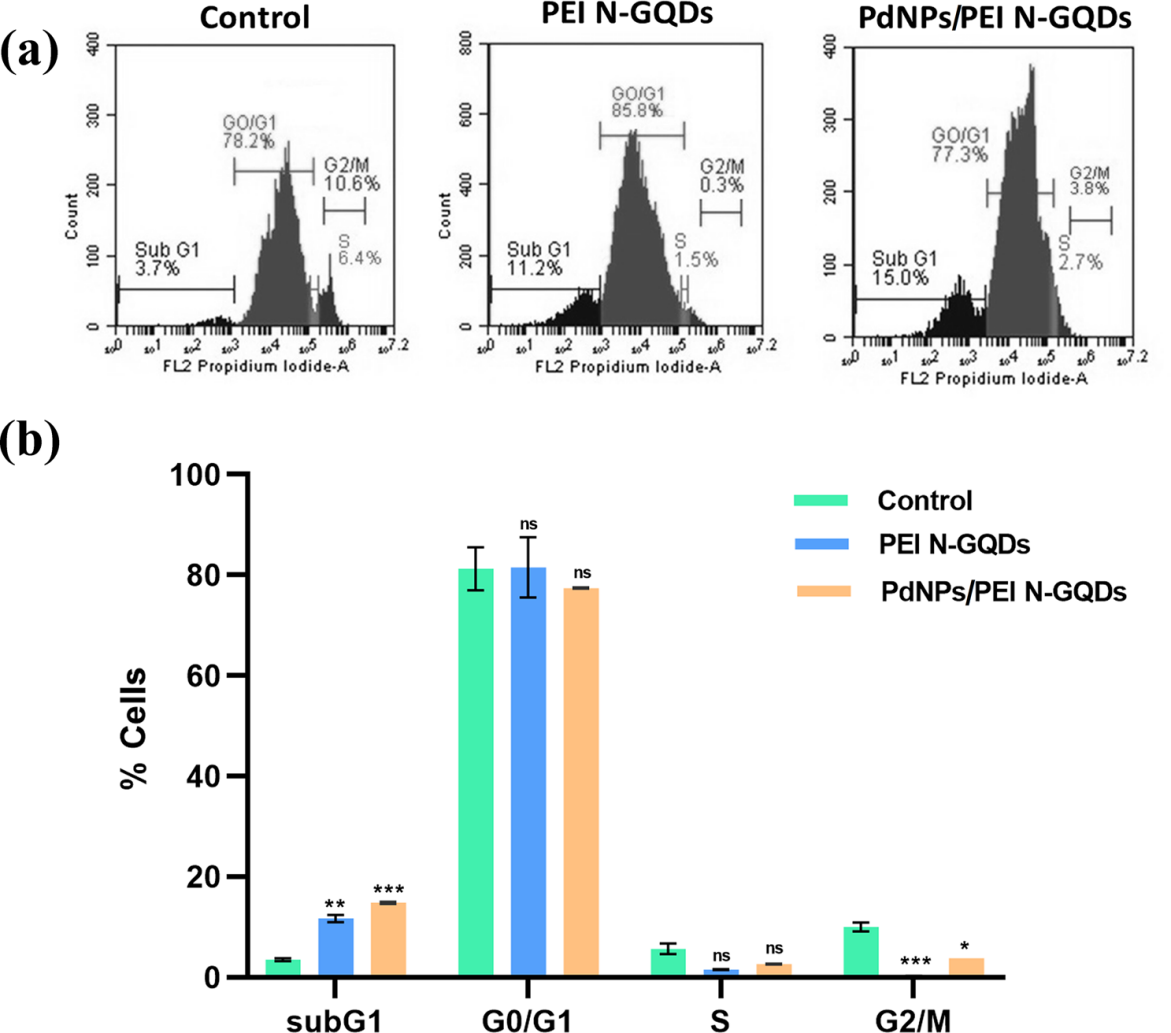
(a) Cell cycle analysis of OVCAR-3 cells treated with nanocomposites was determined by flow cytometry after staining with PI for 48 h; (b) percentages of each cell cycle phase were obtained by flow cytometric analysis (*p < 0.05, **p < 0.001, and ***p < 0.0005 and shows significant differences from control and other groups).

In order to determine the stage at which the cell cycle was arrested in the cells treated with PEI N-GQDs and PdNPs/PEI N-GQDs, we conducted a flow cytometric analysis in our study. Consequently, compared with the untreated control group cells, it was determined to be statistically significant that ovarian cancer cells treated with PEI N-GQDs (*p* < 0.001) and PdNPs/PEI N-GQDs (*p* < 0.0005) were arrested in the sub-G_0_/G_1_ phase of the cell cycle. A statistically significant decrease in the G_2_/M checkpoint of the cell cycle was observed in cells treated with PEI N-GQDs (*p* < 0.0005) and PdNPs/PEI N-GQDs (*p* < 0.05) nanocomposites compared with the control (Figure 12a,b).

According to Tian et al. (2016), GQDs can be genotoxic agents. If this is the case, the genotoxicity will cause the cell to initiate the DNA damage response, which is the process by which the cell stops the progression of the cell cycle in response to DNA damage, giving the lesion enough time to heal.^51^ Ku et al.^52^ reported in their study that GQDs arrested the cell cycle at the G_2_/M checkpoint and induced apoptosis in estrogen receptor-positive breast cancer cell lines. Cell cycle arrest at the sub-G_0_/G_1_ checkpoint is the biggest indicator that chromosomal DNA fragmentation has begun and cells have entered apoptosis.^53^ In response to DNA damage, G_1_/S and G_2_/M checkpoints are triggered to stop cell division in cases in which chromosomes are damaged or absent. Checkpoints for DNA damage allow cells to repair damaged DNA. A damaged DNA molecule can cause a cell to either stop growing or even start dying. Apoptosis is induced by damage to DNA. While the G_1_/S checkpoint stops cells from copying damaged DNA, the G_2_/M checkpoint stops cells from dividing with damaged DNA.^54^ This study provided information about the point at which N-GQD nanocomposites arrest the cell cycle in cancerous cells for the first time in the literature.

Accordingly, it can be concluded that N-GQDs composites cause genotoxic damage to the cell, similar to the literature, and as a result of this, PEI N-GQDs and PdNPs/PEI nanocomposites induce apoptosis by causing chromosomal DNA fragmentation. One may conclude that cell division has ceased at the G_2_/M checkpoint based on the notable drop in the cell population compared with the control. Petrarca et al.^55^ linked a notable increase in cells in the G_0_/G_1_ phase and a noteworthy decrease in the S and G_2_/M phases to the cytotoxicity induced by PdNPs. In contrast, the cancer cells treated with PdNPs/PEI N-GQDs nanocomposites in our investigation were stopped in the sub-G_0_/G_1_ phase, although they still displayed a comparable decline in the G_2_/M checkpoint.

## 4 Conclusions

In this investigation, we conducted a comprehensive exploration involving the synthesis, characterization, and DFT calculation of PEI N-GQDs as well as PdNPs/PEI N-GQDs nanocomposites. Our focus extended to their multifaceted impact on ovarian cancer cells, a pressing concern for women’s health, marked by high mortality rates due to late-stage detection and limited treatment options.

Significantly, we observed that the particle sizes within the PdNPs/PEI N-GQDs nanocomposite were smaller than those in PEI N-GQDs. This size discrepancy, along with the distribution of Pd metal within the nanocomposite, exerted a notable influence on the cell culture outcomes. The smaller particle sizes and the presence of dispersed Pd metal rendered PdNPs/PEI N-GQDs nanocomposites more effective given their enhanced cellular uptake.

Moreover, while PEI N-GQDs reduced Pd(II), they underwent oxidation, transforming into a phenolic structure. This alteration, resulting in the formation of OH groups instead of COOH in the side groups of the molecule, augmented the nanocomposite’s activity. Consequently, PdNPs/PEI N-GQDs nanocomposites exhibited heightened efficacy.

Theoretical investigations revealed stability energies of −3679.098 and −3805.811 hartree for gas-phase-optimized PEI N-GQDs and PdNPs/PEI N-GQDs nanomaterials, respectively. Notably, the PdNPs/PEI N-GQDs nanocomposite emerged as a potential candidate for superior stability, which is a critical factor in optimizing binding affinity and advancing drug design. Additionally, the effects of different PdNP positions within the PdNPs/PEI N-GQD nanocomposite were investigated. A notable interaction is indicated by the observed variations in the dipole moment and HOMO–LUMO energy gap, with respect to the distance from the Pd atom. The identification of a critical threshold points to the ideal configuration for preserving the stability of the electronic structure. Furthermore, the study demonstrated a dynamic interaction impacting the charge distribution, whereby the nanocomposite attained a comparatively stable configuration above a predetermined distance threshold. These findings enhance our understanding of the intricate interplay between PdNPs and the structural and electrical properties of the nanocomposite.

Experimental assessments of cytotoxic, apoptotic, and cell cycle arresting effects on human ovarian cancer cells substantiated the theoretical findings, confirming the enhanced effectiveness of the PdNPs/PEI N-GQDs nanocomposite. The alignment between theoretical and experimental results underscores the reliability of our approach, providing a solid foundation for future studies and projects.

Furthermore, the ability to control and fine-tune the separation between Pd atoms and nanocomposite is of paramount importance. This capability holds significant promise in tailoring electronic and dipole characteristics for specific applications, such as drug design, catalysis, and electronic devices. In summary, our research offers valuable insights into the intricate interplay between nanomaterials and cancer biology, by addressing the pressing need for innovative therapeutic alternatives.

Of particular interest is the integration of PdNPs into ingeniously designed, versatile, and biocompatible N-GQDs with PEI, presenting a compelling fusion at the forefront of materials science. This combination holds the potential to revolutionize the treatment of ovarian cancer by improving the precision in targeting and drug delivery.

## Supporting Information Available

Experimental and theoretical FTIR spectra of the investigated nanocomposites (PDF)
