# Supplementary material for: Palladium Metal Nanocomposites Based on PEI-Functionalized Nitrogen-Doped Graphene Quantum Dots: Synthesis, Characterization, Density Functional Theory Modeling, and Cell Cycle Arrest Effects on Human Ovarian Cancer Cells
Source: ACS Omega. 2024 Mar 6;9(11):13342–58. doi: 10.1021/acsomega.3c10324 (PMC10956410; doi:10.1021/acsomega.3c10324)
Supplement: Supplementary file 1 — ao3c10324_si_001.pdf [file ao3c10324_si_001.pdf]

SUPPLEMENTARY INFORMATION

for

Palladium Metal Nanocomposites Based on PEI-Functionalized Nitrogen-Doped Graphene Quantum Dots: Synthesis, Characterization, Density Functional Theory Modeling, and Cell Cycle Arrest Effects on Human Ovarian Cancer Cells

Buket Altinok Gunes[a,*], Omer Faruk Kirlangic[a], Murat Kilic[a], Asuman Sunguroglu[b], Taner Ozgurtas[c], Ecem Kaya Sezginer[d], Bahadir Boyacioglu[a], Huseyin Unver[e], Mustafa Yildiz[f,*]

[a]Vocational School of Health Services, Ankara University, 06290-Ankara, Türkiye

[b]Department of Medical Biology, School of Medicine, Ankara University, 06620-Ankara, Türkiye

[c]Department of Medical Biochemistry, Gulhane School of Medicine, University of Health Sciencies, 06018-Ankara, Türkiye

[d]Department of Biochemistry, Faculty of Pharmacy, Ankara University, 06100-Ankara, Türkiye

[e]Department of Physics, Faculty of Science, Ankara University, 06100-Ankara, Türkiye

[f]Department of Chemistry, Faculty of Sciences, Canakkale Onsekiz Mart University, 17100-Canakkale, Türkiye

[*]Corresponding Author(s): baltinok@ankara.edu.tr (B.A. GUNES),
myildiz@comu.edu.tr (M. YILDIZ)

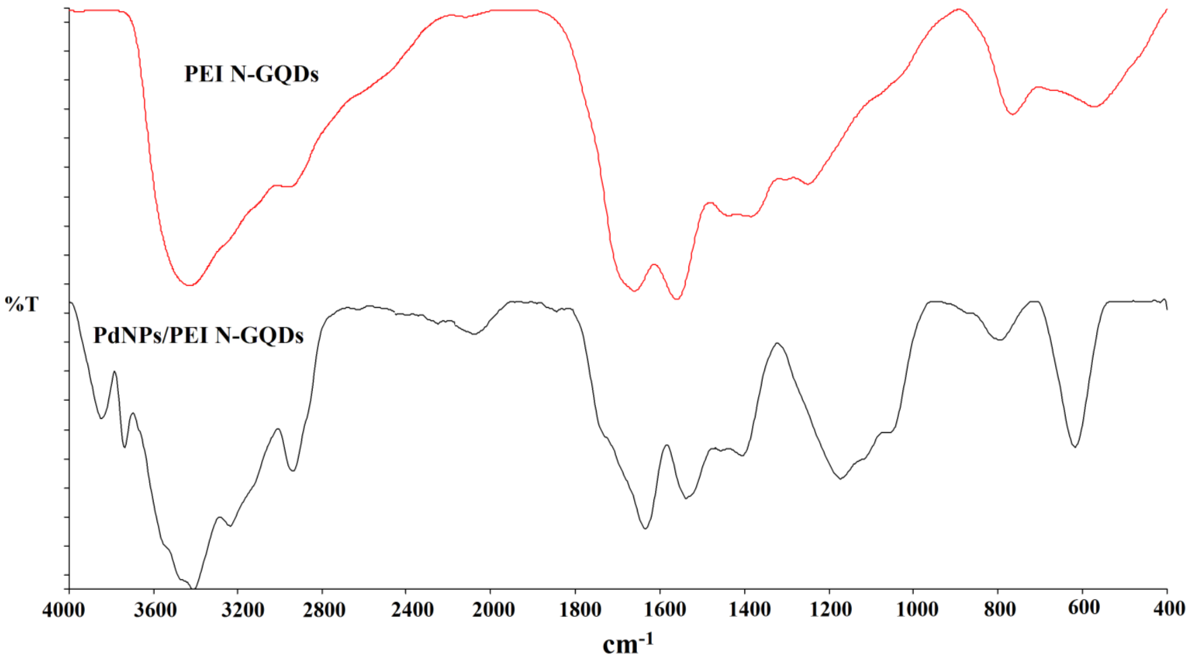

Figure S1. The experimental FTIR specta of the investigated nanocomposites

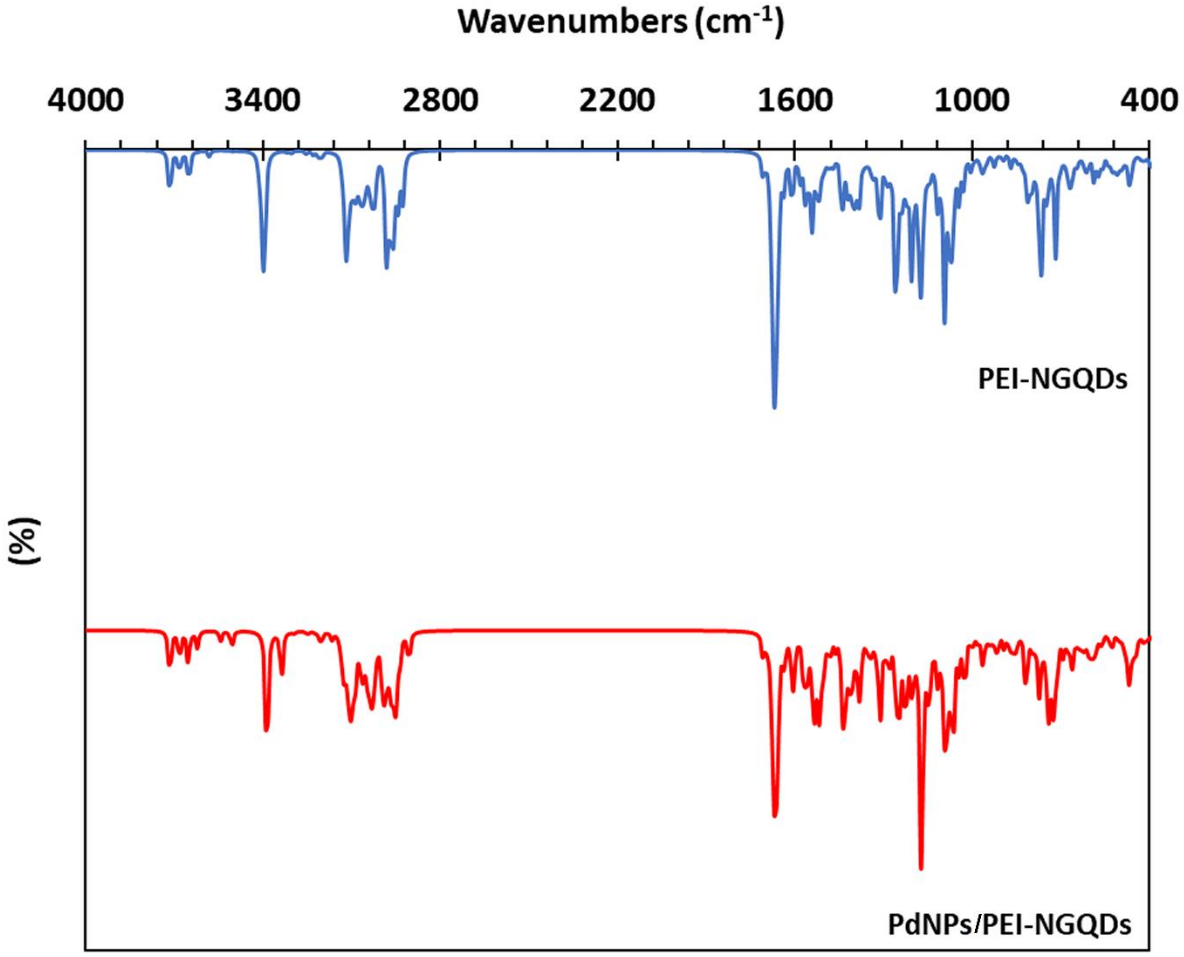

Figure S2. The theoretical FTIR specta of the investigated nanocomposites
